# From Gas to Solution: The Changing Neutral Structure of Proline upon Solvation

**DOI:** 10.1021/acs.jpca.4c05628

**Published:** 2024-11-13

**Authors:** Bruno Credidio, Stephan Thürmer, Dominik Stemer, Michele Pugini, Florian Trinter, Jakub Vokrouhlický, Petr Slavíček, Bernd Winter

**Affiliations:** † Molecular Physics, Fritz-Haber-Institut der Max-Planck-Gesellschaft, Faradayweg 4-6, Berlin 14195, Germany; ‡ Department of Chemistry, Graduate School of Science, Kyoto University, Kitashirakawa-Oiwakecho, Sakyo-Ku, Kyoto 606-8502, Japan; § Department of Physical Chemistry, University of Chemistry and Technology, Technická, Prague 16628, Czech Republic

## Abstract

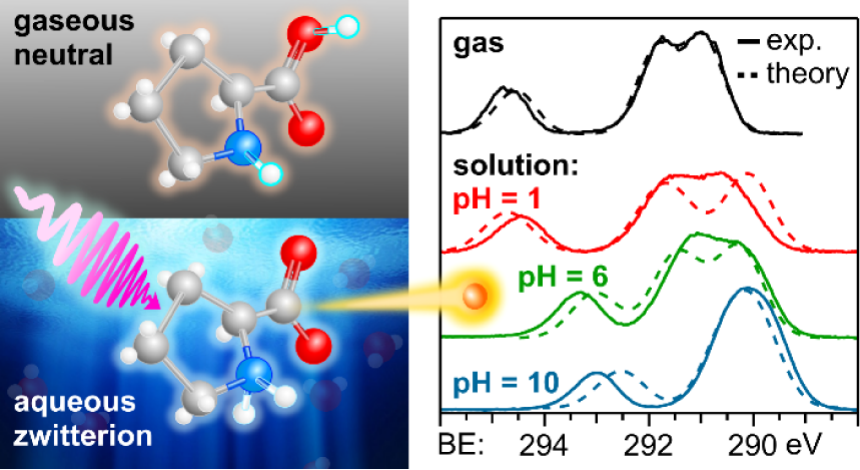

Liquid-jet photoelectron spectroscopy (LJ-PES) and electronic-structure theory were employed to investigate the chemical and structural properties of the amino acid l-proline in aqueous solution for its three ionized states (protonated, zwitterionic, and deprotonated). This is the first PES study of this amino acid in its biologically relevant environment. Proline’s structure in the aqueous phase under neutral conditions is zwitterionic, distinctly different from the nonionic neutral form in the gas phase. By analyzing the carbon 1s and nitrogen 1s core levels as well as the valence spectra of aqueous-phase proline, we found that the electronic structure is dominated by the protonation state of each constituent molecular site (the carboxyl and amine groups) with small yet noticeable interference across the molecule. The site-specific nature of the core-level spectra enables the probing of individual molecular constituents. The valence photoelectron spectra are more difficult to interpret because of the overlapping signals of proline with the solvent and pH-adjusting agents (HCl and NaOH). Yet, we are able to reveal subtle effects of specific (hydrogen-bonding) interaction with the solvent on the electronic structure. We also demonstrate that the relevant conformational space is much smaller for aqueous-phase proline than for its gas-phase analogue. This study suggests that caution must be taken when comparing photoelectron spectra for gaseous- and aqueous-phase molecules, particularly if those molecules are readily protonated/deprotonated in solution.

## 1 Introduction

Proline (pyrrolidine-2-carbonic acid) distinguishes itself within the proteinogenic amino acids by its heterocyclic amine group, which imparts higher rigidity and reduces its conformational space. It plays a crucial role in collagen synthesis, a major structural protein in the human body, and is involved in various cellular processes, including gene expression, cell signaling, redox reactions, and stress protection.^1−5^ The availability of proline significantly affects collagen synthesis, with glutamine being a key regulatory amino acid in this process.^6^ Understanding the behavior of proline in aqueous solutions is essential for elucidating its properties in biologically relevant environments such as the interior of cells. For example, hydration effects in amino acids are known to play an important role in processes such as protein folding and enzyme function.^7−9^

Proline’s predominant neutral form in the aqueous phase is different than in the gas phase. At close to neutral pH in water (similar to physiological conditions), proline exclusively adopts a zwitterionic form, stabilized by hydrogen bonding with water molecules. It has been shown that even a single water molecule can stabilize this form of proline, highlighting the significant role of hydration on its structural stability.^7^ In contrast, zwitterionic proline does not exist in the gas phase, where the molecule is in its nonionic neutral form instead. This discrepancy in the charge state of neutral amino acids in the condensed versus gas phase has long been known,^10^ and implications for the relevance of gas-phase studies for understanding the solution-phase electronic structure of biologically relevant molecules have been described in a pioneering study on aqueous-phase glycine.^11^

**Figure 1:**
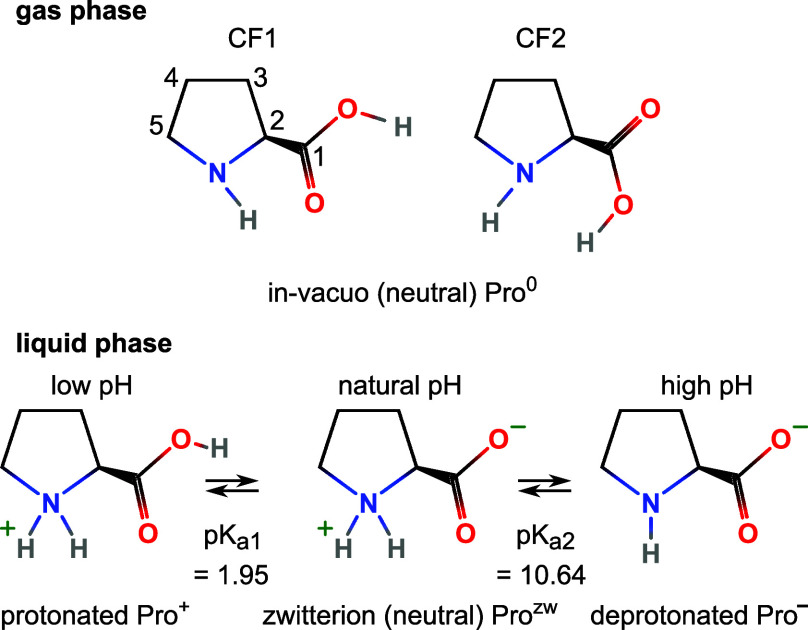
Sketches of the proline molecule: (Top) gas phase, Pro^0^, in its two possible conformers CF1 and CF2, which are each composed of two energy-degenerate forms due to ring puckering.^17^ (Bottom) aqueous phase: Pro^+^, Pro^zw^, and Pro^–^. p*K*_a_ values are taken from ref. (19).

The different protonation forms of proline are summarized in Figure 1, where we concentrate on the two primary protonation sites, the heterocyclic nitrogen and the carboxylic group. In the gas phase, both sites are singly protonated, giving the molecule overall charge neutrality (Pro^0^). In the aqueous phase, the neutral zwitterionic (Pro^zw^) form instead features a doubly protonated nitrogen in the ring (excess positive charge) and a deprotonated carboxylic group (excess negative charge). It can be expected that these structural differences have a large impact on the molecule’s electronic structure, with considerable implications for its molecular function. Protonation/deprotonation is difficult to realize in the gas phase. However, in aqueous solution, the protonation state can be readily changed by pH, which produces protonated (positive, Pro^+^) proline at low pH and deprotonated (negative, Pro^–^) proline at high pH; compare Figure 1 for the respective p*K*_a_ values. Noticeably, for both gas-phase Pro^0^ and aqueous-phase Pro^+^, the carboxylic group is protonated, while Pro^0^ and Pro^–^ both have a singly protonated amine group. The amine and carboxyl protonation states will thus be important when comparing the remarkably different electronic structure of the neutral states in the respective phases, which is analogous to glycine,^11^ and similar conclusions can be drawn here.

A second aspect we will explore here is the existence of different conformers of proline. This is different from the dynamical range of hydration configurations in real liquid water due to fluctuations in the hydrogen-bond pattern (based on snapshots from the MD simulations), which was discussed for glycine to explain the associated N 1s and C 1s core-level PES spectral widths.^11^ In the present case of proline, different conformers occur in the gas phase with considerably different N 1s core-level and valence spectra, giving rise to bimodal distributions. However, the conformer space strongly reduces in the aqueous phase, which can severely complicate an assignment of the aqueous-phase valence and C 1s PES spectra based on the gas-phase spectrum, as we will discuss. Previous gas-phase studies have employed electron diffraction, microwave spectroscopy, and quantum chemical calculations to identify the four most stable conformers of Pro^0^, which are stabilized by intramolecular hydrogen bonds and associated differences in ring puckering and carboxylic-acid orientation.^12^ Those results have been confirmed by subsequent gas-phase photofragmentation and argon-matrix infrared-spectroscopy experiments.^13,14^ The conformers are differentiated primarily by the rotational angle of the carboxylic acid with respect to the amine groups, with each rotation (OH toward and away from the amine) exhibiting two nearly energetically degenerate conformations that differ only via small changes in ring puckering. The energetic differences associated with ring puckering are very small and cannot be resolved experimentally by photoelectron spectroscopy (PES). Hence, we consider only two conformer classes, completely described by the orientation of the carboxylic acid relative to the amine group, as sketched in Figure 1, top, adopting the CF1 and CF2 labeling from ref. (15). Plekan et al. have used PES to determine proline’s carbon 1s and nitrogen 1s core-level energies, as well as the respective valence energies in the gas phase.^16,17^ A clear signature from these different conformers was revealed from the nitrogen 1s and the valence spectra, not from the carbon 1s spectra. Additional higher-energy conformers can be expected,^14,18^ however, with low populations at room temperature, and are not considered further. How the spectroscopic signature of the two prevalent gas-phase conformers helps assign the corresponding pH-dependent aqueous-phase photoelectron (PE) spectra is one central aspect of the present study.

The structure of proline in the aqueous phase has not yet been explored at the same level of detail due to the lack of appropriate experimental tools. It is noted though that the conformational distinction between CF1 and CF2 is rather irrelevant in the aqueous phase in the case of deprotonation of the carboxylic group; compare Figure 1, bottom. This fact is important to be aware of when comparing gas- and aqueous-phase PE spectra. Previously, resonant inelastic soft X-ray scattering (RIXS) and X-ray emission spectroscopy (XES) from the nitrogen and oxygen edges using liquid-flow cells have provided electronic-structure information on proline in aqueous solutions at low (0.8), intermediate (6.8), and high (13.0) pH,^20^ i.e., for proline’s three distinct protonation states depicted in Figure 1. It has been reported that the electronic structure is dominated by the protonation state of the amine and carboxylic acid groups and can be directly related to the analogous structural building blocks, with pyrrolidine being representative of the heterocyclic amine and acetic acid of the carboxylic acid. Our results will be shown to confirm this observation and additionally provide accurate site-specific electron binding energies for all relevant proline species in the aqueous phase, as well as identifying prevalent conformers. Furthermore, distinct features in the nitrogen spectra have been assigned to the five-membered ring structure of the molecule.^20^ In a related context, Saykally et al. have also characterized proline aqueous solutions with total electron yield X-ray absorption spectroscopy, both at neutral and high-pH conditions, utilizing liquid microjets;^21^ the latter is also used in the present work. Specifically, the nitrogen K-edge of aqueous proline has been measured, revealing structural specifics of the hydration of the nitrogen terminus.

Here, we employ liquid-jet photoelectron spectroscopy (LJ-PES) and well-tested electronic-structure calculations, capable of probing the electronic structure and solvation dynamics of biomolecules in their natural aqueous environment.^22−25^ Specifically, the present study explores the core-level and valence electronic structures of aqueous-phase Pro^+^, Pro^zw^, and Pro^–^. We quantify and discuss the occurring energetic changes upon pH variation, and demonstrate the limited conformational space compared to the gas phase. This work is also part of our wider goal to establish empirical rules for the interpretation of the liquid-phase photoelectron spectra.^24^

## 2 Methods

### 2.1 Experimental Section

Measurements were carried out at the soft X-ray beamline P04 of the PETRA III synchrotron facility, Deutsches Elektronen-Synchrotron (DESY, Hamburg, Germany),^26^ using our state-of-the-art LJ-PES setup *EASI* (Electronic structure from Aqueous Solutions and Interfaces).^27^ The spectrometer is equipped with a near-ambient-pressure hemispherical electron analyzer (Scienta-Omicron HiPP-3). Differential pumping stages ensure sufficiently low pressures in both the spectrometer and the beamline. Efficient μ-metal shielding ensures magnetic-field-free conditions in the interaction region, where the X-ray beam crosses the LJ, both propagating in the horizontal (floor) plane and perpendicular to each other. The electron detection direction is at an angle of 130° with respect to the light propagation direction (backward-detection configuration) and normal to the LJ, which was situated ∼0.8 mm away from the 0.8 mm skimmer orifice of the spectrometer. During the experiments, the pressure inside the interaction chamber was kept at ∼5 × 10^–4^ mbar by two turbomolecular pumps with a combined pumping speed of ∼2600 L/s for N_2_, and three liquid-nitrogen-cooled traps with a total pumping speed of ∼35000 L/s for water. The LJ was frozen and collected at one of these traps situated at the far end of the interaction chamber.

We have used a pass energy of 50 eV and an entrance slit of the hemisphere of 0.8 mm, yielding a kinetic-energy (KE) resolution of ∼100 meV. The undulator at beamline P04 provides circularly polarized light in the range of 250–3000 eV. We have used photon energies in the range of 270–500 eV, which were selected by a 1200 lines/mm laminar grating. The vertical exit slit of the beamline was set to 150 μm, which yields a beam focus of 180 μm (horizontal) × 50 μm (vertical) at the microjet; the latter value is relevant for maximizing the spatial overlap with and was only slightly larger than the LJ, which has a diameter of ∼25–35 μm.

The carbon 1s and valence-band spectra were measured with a photon energy of 379.66 ± 0.08 eV in a first campaign, where we also measured nitrogen 1s spectra at 499.47 ± 0.08 eV photon energy. The photon-energy calibration was conducted by measuring the energy offset to known absorption bands of nitrogen, argon, and nitrogen. This method is not as precise as using a photon energy at a reference absorption feature directly (see below), but the relative peak positions are expected to be correct within ±0.05 eV. The C 1s and valence-band measurements were repeated in a second campaign, where photon energies were calibrated by using a value very close to the N 1s π* transition of nitrogen gas, measured in a dedicated analysis chamber that is part of the beamline,^28^ which yielded a precise photon energy of 403.08 ± 0.03 eV. Both campaigns yielded practically identical results for the purpose of the current study; see SI for details.

**Figure 2:**
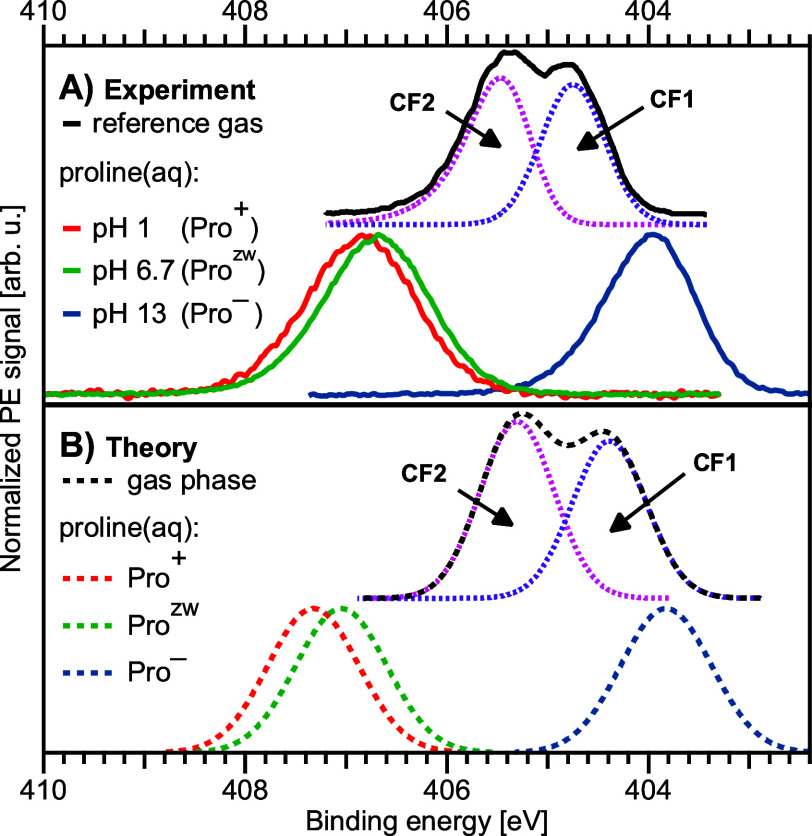
A) N 1s spectra of 1 M proline(aq) in the protonated (pH 1, red), zwitterionic (pH 6.7, green), and deprotonated (pH 13, blue) states measured at a photon energy of 499.47 eV. 10% signal contribution of the zwitterionic species was subtracted from the spectrum of the protonated species (see Methods and SI for details). The gas-phase spectrum from Plekan et al.,^16^ measured at 495 eV photon energy, is plotted in black at the top. A split peak is observed, originating from the two conformers CF1 and CF2 in the gas phase with slightly different BEs,^16^ here indicated by two contributions (dotted lines) as a guide to the eye, which are approximated with a Gaussian (CF1) and Exponentially Modified Gaussian (CF2) peak shape, respectively. B) Corresponding calculated spectra shown as dashed lines, where the spectra of the protonated (red), zwitterionic (green), and deprotonated (blue) species have been shifted by −0.42, 0.24, and 0.79 eV, respectively, analogous to Figure 3; these shifts were extracted from the C 1s spectral comparison. The gaseous contributions of conformers CF1 and CF2 have been added in the same 1:1.12 ratio as in the experiment.

**Figure 3:**
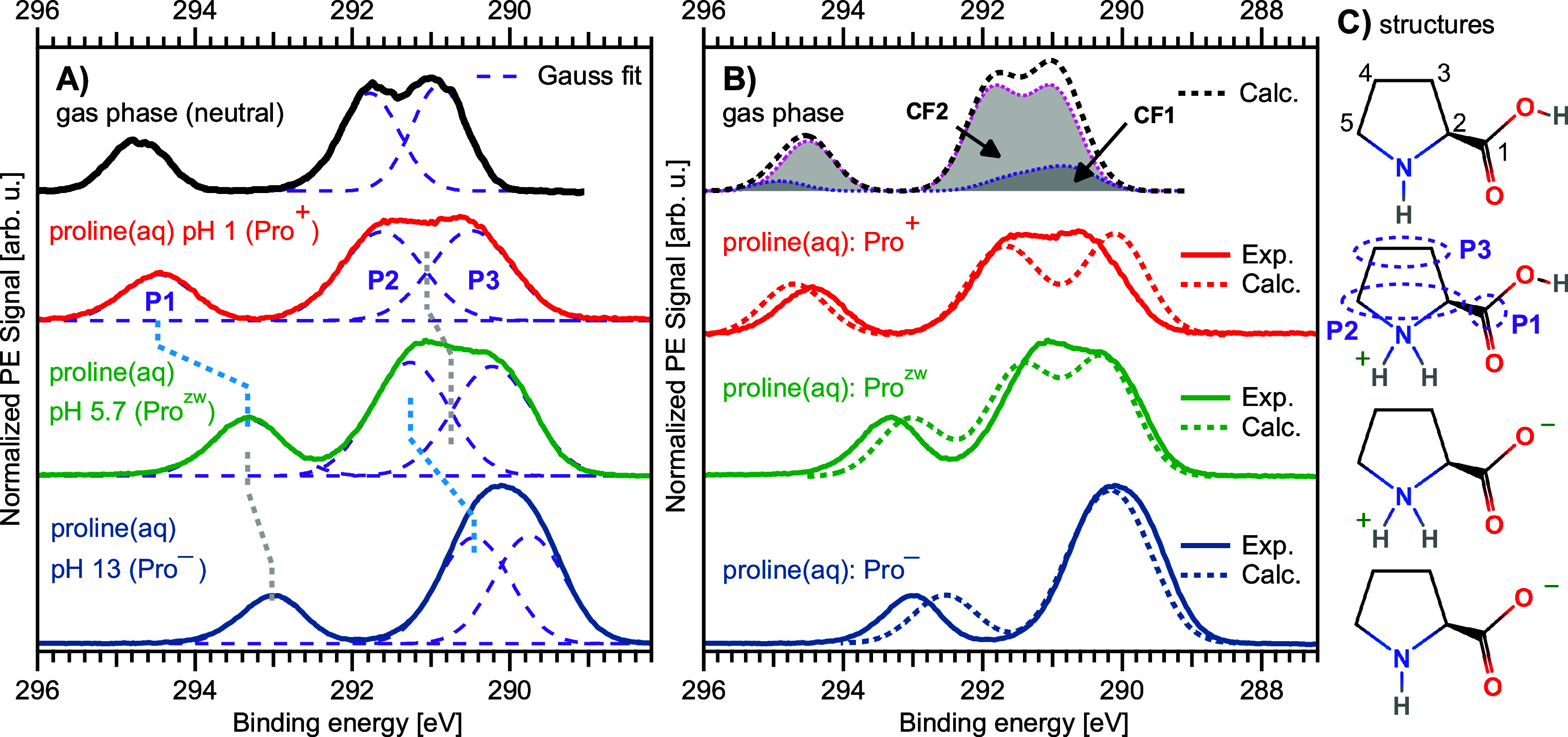
A) C 1s spectra of 1 M proline(aq) in its protonated (pH 1, red), zwitterionic (pH 5.7, green), and deprotonated (pH 13, blue) state. A 10% signal contribution of the zwitterionic species was subtracted from the spectrum of the protonated species (see Methods and SI for details). The spectrum in black is from gaseous proline from ref. (16). Purple dashed lines are Gaussian fits to the experimental spectra, which were constrained to yield equal areas for peaks P2 and P3. B) Liquid-phase experimental spectra in comparison to their theoretical counterparts. The theoretical spectra of the protonated (dotted red), zwitterionic (dotted green), and deprotonated (dotted blue) species have been shifted by −0.42, 0.24, and 0.79 eV, respectively; this matches the centroid of each spectrum with the experimental one and is compensating for an over/under-estimation of the polarization screening in the model (these are the same shifts applied to the N 1s theory in Figure 2). The spectrum in black at the top is a computation of the neutral molecule in the gas phase, which consists of contributions from the two conformers CF1 and CF2 as indicated by the labels; both contributions were summed with a ratio of 1:4, which does not match that found in the nitrogen data but best fits the experimental gas-phase spectrum. C) A sketch of the molecule in each respective state (reproduced from Figure 1) is shown next to the corresponding spectra; we enumerate each carbon site in the topmost sketch. The labels P1, P2, and P3 are the three spectral features from the aqueous phase, as introduced in the main text, along with their correspondence to the molecule’s carbon atoms as indicated in one of the molecular sketches by the purple ovals.

The LJ was formed by a quartz nozzle with a 34.6 μm orifice, to which the sample was delivered with a flow rate of 0.8 mL/min via a Shimadzu LC-20AD high-performance liquid chromatography (HPLC) pump equipped with a degasser (Shimadzu DGU-20A5R). The LJ assembly features a water-cooled jacket that provides thermal control; the temperature was set to 10 °C in the chiller unit. The temperature at the interaction point situated a few millimeters downstream from the nozzle is expected to have a somewhat lower temperature due to evaporative cooling. The aqueous solutions were prepared by mixing l-proline (Sigma-Aldrich, ≥ 99% purity) into demineralized water (conductivity ∼0.2 μS/cm) to yield a 1 M concentration. To adjust the solution’s pH, we added hydrochloric acid (to yield pH 1) or sodium hydroxide (to yield pH 13), assuring to keep a 1 M proline concentration. For the zwitterionic solution (pH 5.7), 50 mM of sodium chloride was added to ensure sufficient electrical conductivity and to avoid charge-up of the microjet. In the first measurement campaign, the pH value of the zwitterionic solution was adjusted to 6.7, which is, however, far enough from either p*K*_a_ value (see Figure 1) to be irrelevant for the core-level results. Note also that pH = 1.0 is only one unit below p*K*_a1_ (compare Figure 1); solutions with pH < 1.0 did not provide stable experimental conditions. For that reason, the measured Pro^+^ spectrum contains a small, approximately 10% contribution from Pro^zw^, which, however, has been subtracted in Figures 2 and 3; the procedure is detailed in the Supporting Information. For the two other spectra, single-species populations, Pro^zw^ and Pro^–^, respectively, can be assumed.^29^

Electronic binding energies (BEs) were determined using the recently established absolute energy referencing scheme.^22,30−32^ Briefly, a metallic connector in the liquid delivery line is used to apply a negative bias voltage to the liquid jet, which shifts the PE spectrum toward higher KEs. This reveals the low-energy tail (LET) and low-energy cutoff of the spectrum, the latter identifying zero KE, i.e., the onset of a corrected KE scale of electrons after their escape from the liquid surface into vacuum. We obtain absolute BEs of any solute or solvent species from the energy distance of the respective PE peaks to the cutoff, i.e., KE_corr_, and the precisely known photon energy (*h*ν) via BE = *h*ν – KE_corr_. Notably, the new method applied here does not refer to the energetic distance between gas- and liquid-phase peaks, which is an ill-defined quantity due to multiple charging effects of the liquid jet.^22,30−32^ Moreover, the application of a bias voltage implies that electrons from ionization of gas-phase water molecules have different KEs, depending on the distance of the water molecules from the jet surface, sensing the electric field between the liquid jet and the electron detector. In the case of sufficiently large (approximately −50 V) bias voltage this spreads out the gas-phase signal to an extent that the associated signal only results in a shallow background underneath the aqueous-phase PE spectrum.^22,30−32^

In the present study, we have not attempted to explore explicit properties of the solution–vacuum interface, such as possible changes in surface activity depending on the charge state or ion pairing at the 1 M concentration. We note that for all solutions, Pro^+^(aq), Pro^zw^(aq), and Pro^–^(aq), concentration considerably larger than 1 M (up to 14 M at room temperature)^33^ may be obtained, indicating proline’s high solubility in water. We also note that proline does not exhibit hydrophobic functional groups and is rather known to strongly interact with water, further indicating that surface effects are likely to play only a negligible role in the context of the present work (see also the discussion of the C 1s spectra).

### 2.2 Calculations

The structure of proline was estimated in its three protonation states, distinguishing the zwitterionic and neutral forms. The optimization was performed with the wB97XD^34^ functional using the 6–31+g* basis set. The optimization was done in a dielectric continuum, represented by a polarizable continuum model (PCM),^35,36^ using the standard atomic radii within the universal force field (UFF) and an electrostatic scaling factor α = 1.1. The optimization in the dielectric continuum was needed, as the zwitterion is unstable in the gas phase. Cartesian coordinates of the optimized structures can be found in the Supporting Information.

The core-level binding energies were estimated with the maximum-overlap method (MOM),^37^ using its augmented form (initial maximum-overlap method) to achieve a better convergence.^32^ The technique is based on a selection of ionized orbitals. A regular self-consistent field procedure is then performed with additional Lagrange multipliers to avoid the variational collapse of the wave function. We have used here the Coulomb-attenuating method based on the B3LYP functional (CAM-B3LYP),^38^ combined with the aug-cc-pVTZ basis set for hydrogen atoms and the aug-cc-pCVTZ basis set^39,40^ for carbon, oxygen, and nitrogen atoms. Only the binding energies of the carbon and nitrogen atoms were calculated since the oxygen signal strongly overlaps with the solvent. This combination provides consistent and accurate binding energies with an error of several tenths of eV.^41^ The solvent response during the electron ejection was modeled with the nonequilibrium PCM model that separates the response into the fast (optical) response and the slow (nuclear) response.^42,43^ Only the former is considered during the ionization process. The application of the nonequilibrium model is critical to achieve accurate binding energies in the liquid phase.

The valence electron binding energies were calculated with a combined approach. The binding energy of the highest occupied molecular orbital (HOMO) was estimated by using the same approach as the core-level binding energies. As the electron levels are closely spaced in the valence domains, it was not possible to stabilize them by the MOM method. We have, therefore, calculated the higher binding energies by calculating the HOMO energy level first, adding then the excitation energy of the nascent radical cation to estimate the HOMO-*n* levels. The time-dependent density functional theory (TDDFT) was used for these calculations, again using the CAM-B3LYP functional. This approach was previously shown to provide reliable valence photoelectron spectra in the liquid phase.^42^

The photoelectron spectra were constructed from the calculated binding energies using the empirical broadening scheme. Each binding energy value was broadened with a Gaussian function of the same intensity and width, where the full width at half maximum (FWHM) was 0.87 eV for the gas phase and 1.06 eV for the liquid phase. These width values match the FWHM of the P1 peak of the experimental C 1s spectrum in each case. The width was selected based on previous experience with analogical systems, and it accounts for lifetime broadening, vibrational broadening, and inhomogeneous broadening due to the solute–solvent interactions for the liquid-phase calculations. The Gaussian form turns out to be reasonable, even for the core-excited states.

All the calculations were performed in the Q-Chem package,^44^ version 6.0 except for the TDDFT calculations where Gaussian 09, rev. D.01 was used.^45^

## 3 Results

### 3.1 Core-Level Spectra: A Direct Structural Probe

We start the discussion of the structural assignment for the different protonation states of proline with core-level spectra, which are the most sensitive structural probes. The nitrogen 1s PE spectrum is easiest to interpret, as there is only a single nitrogen atom in the molecule. The carbon 1s spectrum is more complicated, with strongly overlapping intensities from the different carbon sites. In principle, oxygen 1s spectra could be recorded as well, yet the PE signal would be completely dominated by the water solvent contribution.

#### 3.1.1 N 1s PE Spectra

With reference to Figure 2, we begin the discussion with the experimental (Figure 2A) and the computed (Figure 2B) nitrogen 1s core-level PE spectra that show the clearest evidence for the electronic structural variations between gas-phase and aqueous-phase proline. The liquid-phase spectra were measured from solutions at 1 M concentration for three different pHs, 1.0 (red), 6.7 (green), and 13.0 (blue); the photon energy was 499.47 eV. Based on the known p*K*_a_ values for proline (Figure 1), we expect that these values correspond to the Pro^+^, Pro^zw^, and Pro^–^ structures; a small signal contribution of Pro^zw^ has been subtracted from the Pro^+^ spectrum (see Methods). These were also the structures used for the calculations, and we present the corresponding core-level spectra for the Pro^+^, Pro^zw^, and Pro^–^ structures in Figure 2B. The calculated liquid-phase spectra were shifted as indicated in the figure caption; these shifts are further clarified later when discussing the carbon 1s spectra.

The comparison between theory and experiment supports the correctness of the structural assignment for the given pHs. Both the protonated and the zwitterionic proline exhibit rather similar N 1s BEs, approximately 407 eV, whereas the respective energy for the deprotonated form is lower by more than 2.7 eV, near 404 eV; numerical values are shown in Table 1. This large difference is a direct effect of the protonation with the positive charge stabilizing the core electron. The only slightly larger BE for Pro^+^ (by 0.15 eV) compared to Pro^zw^ implies that the core-level electron BEs are to a large extent controlled by the local chemical environment. The influence of the charged carboxylic group in the zwitterion is rapidly attenuated due to the dielectric effect from polarization of nearby water molecules and thus has a very small measurable effect on the BE.

**Table 1:** Experimental (Top) and Theoretical (Bottom) N 1s Binding Energies^a^

	State	CF1 [eV]	CF2 [eV]	CF_liq_ [eV]
gas (exp)^16^	Pro^0^	404.8	405.5	-
liquid (exp)	Pro^+^	-	-	406.84
liquid (exp)	Pro^zw^	-	-	406.68
liquid (exp)	Pro^–^	-	-	403.96
gas (theory)	Pro^0^	404.4	405.3	-
liquid (theory)	Pro^+^	-	-	407.32
liquid (theory)	Pro^zw^	-	-	407.05
liquid (theory)	Pro^–^	-	-	403.82

a The liquid-phase theory value has been shifted by −0.42, 0.24, and 0.79 eV for the protonated (red in Figure 2), zwitterionic (green), and deprotonated (blue) species, respectively.

The calculated data almost quantitatively reproduce the experiment on an absolute scale after application of energy shifts for compensating screening effects; these shifts were determined by matching the experimental and theoretical centroids in the C 1s spectra. The remaining energetic discrepancy of several tenths of eV to the experiment is attributed to the limited accuracy of the density functional technique used for the calculations. However, experimental details, such as the small energetic shift between the protonated and zwitterionic species, are well reproduced.

We now contrast the measured Pro^zw^ spectrum with its gas-phase analogue as measured previously by Plekan et al.^16,17^ There are two structural differences in the gas phase. First, the gas-phase molecule appears in the Pro^0^ form, with both nitrogen and carboxyl singly protonated. Second, as aforementioned, two dominating groups of conformers contribute to the spectrum; namely CF1 and CF2 as shown in Figure 1. The experimental spectra from ref. (16) and our calculated spectra are shown in Figure 2 at the top of panels A and B, respectively. Plekan et al. observed two well separated peaks that were attributed to CF1 and CF2, as indicated, with a ratio of 1:1.12, i.e., the CF2 conformer has a slightly higher abundance;^16^ we present the two signal contributions as two simple peak shapes, detailed in the figure caption, with the same ratio as a guide to the eye in Figure 2A. We consider the same CF1 and CF2 structures for our calculations, i.e., a CF1 conformer with the hydrogen of the carboxylic group positioned opposite from the nitrogen atom, and a CF2 conformer with the bound hydrogen atom located between the COOH and NH groups (Figure 1). The latter interaction leads to stabilization of the electron in the nitrogen, leading to a 0.7 eV higher BE; the calculation reproduces this shift well (Figure 2, bottom).

As discussed earlier, these conformers are not possible for the deprotonated and zwitterionic species of proline with a deprotonated carboxylic group; CF1 and CF2 represent different orientations of the hydrogen within the COOH group, which is absent in Pro^zw^ and Pro^–^. Nevertheless, we used computational tools to investigate the electronic structure of the hypothetical solvated Pro^0^ CF1 and CF2 conformers. The theoretical spectrum of Pro^0^ in the aqueous phase has a shape similar to that of the gas phase, with the N 1s peaks corresponding to CF1 and CF2 appearing at binding energies 1.5–2.0 eV below those experimentally measured for Pro^zw^ in solution (additional details may be found in Figure S1), further indicating that the gas-phase CF1 and CF2 conformers do not appear to be relevant for understanding the structure of aqueous-phase proline. Above-mentioned calculations and the strong similarity between the Pro^zw^ and Pro^+^ N 1s spectra do not indicate that the isomerism in the gas phase is significant in solution. In other words, different major structural conformers with distinct intramolecular hydrogen bonding do not appear to be significant in the aqueous phase.

#### 3.1.2 C 1s PE Spectra

The analysis of the carbon 1s PE spectrum is complicated by multiple carbon-atom sites. Figure 3 summarizes all relevant experimental and computed gas- and aqueous-phase C 1s spectra of proline. Figure 3A, top, shows the Pro^0^ spectrum, reproduced again from Plekan et al.^16^ Below that, from top to bottom, we present the spectra measured from 1 M proline(aq), at pH = 1.0 (red), 5.7 (green), and 13.0 (blue), corresponding to Pro^+^, Pro^zw^, and Pro^–^. Note that the N 1s PES measurements of Pro^zw^ shown in Figure 2 have been performed at the slightly adjusted pH of 6.7, whereas the respective C 1s measurements from Figure 3 were conducted at the solution’s natural pH of 5.7. This small difference has no effect for the present purpose, being several pH units separated from both p*K*_a_ values, and thus assuring Pro^zw^ as the only species. Purple dashed lines are the respective Gaussian fits to extract the electron BEs of the different carbon atoms (atom groups). They are labeled as shown in Figure 3C, where we provide the respective sketches of the proline structures Pro^0^, Pro^+^, Pro^zw^, and Pro^–^; for Pro^0^, only the CF1 conformer is shown.

All spectra exhibit a similar overall structure with one smaller isolated band (P1) at higher BEs and two larger overlapping bands (P2 and P3) at lower BEs; the corresponding BE values are summarized in Table 2. A first qualitative assignment of these bands to the five carbon atoms of proline is rather straightforward, judged from the chemical shift expected from the electronegativity of the (changing) local environment. Accordingly, P1 can be associated with the carbon atom of the carboxylic group; its larger BE reflects the partial withdrawal of electron density from the carbon atom to the strongly electronegative oxygen atoms which makes the C1 carbon site more positive. Peak P2 contains contributions from C2 and C5, and peak P3 from C3 and C4. The BE of peak P2, intermediate between peaks P1 and P3, can be qualitatively attributed to the vicinity to the nitrogen, which also draws electron density from the carbons but is less electronegative than oxygen.

**Table 2:** Experimental (Top) and Theoretical (Bottom) C 1s Binding Energies^a^

	State	P1 [eV]	P2 [eV]	P3 [eV]
gas (exp)^16^	Pro^0^	294.7	291.8	290.9
liquid (exp)	Pro^+^	294.47	291.62	290.49
liquid (exp)	Pro^zw^	293.33	291.26	290.22
liquid (exp)	Pro^–^	293.02	290.45	289.75
gas (theory)	Pro^0^	294.71	291.65	290.82
liquid (theory)	Pro^+^	294.68	291.65	290.08
liquid (theory)	Pro^zw^	293.03	291.46	290.26
liquid (theory)	Pro^–^	292.53	290.49	289.89

a The liquid-phase theory has been shifted by −0.42, 0.24, and 0.79 eV for the protonated (red in Figure 2), zwitterionic (green), and deprotonated (blue) species, respectively, after averaging over each carbon contribution for the carbon groups contributing to P2 and P3, respectively. The theoretical gas-phase values represent an average over each carbon contribution for the carbon groups contributing to P2 and P3, respectively, as well as conformers CF1 and CF2. Individual values for each carbon atom are summarized in the SI.

We now provide a more detailed description of the observed spectral changes associated with both proline’s net molecular charge and local protonation state. The first observation is a global shift of the whole C 1s spectrum when moving from the protonated species to the deprotonated species. The core-level electrons of the protonated species are (on average) bound most strongly, while the ones within the deprotonated, negatively charged proline are bound the least. This change is easy to understand as resulting from Coulombic attraction (Pro^+^) or repulsion (Pro^–^). The energy shift is, however, relatively small (1.0 eV shift toward lower BEs of the spectral centroid) due to the strong screening by the water solvent.^42^ It is interesting to look at the total shift of the spectrum between the gas-phase Pro^0^ and its neutral analogue Pro^zw^ in the aqueous phase, which is 0.77 eV. This is typical for gas–liquid shifts for organic molecules in aqueous solution.,^22,30,46^

The change of (local) protonation state, on the other hand, should lead to a relative shift mainly of the band associated with the protonated/deprotonated atomic site, which causes a redistribution of charge density. Specifically, we expected that the change in energy of peak P1 (associated with the carboxylic group) should be the largest when crossing p*K*_a1_. Analogously, the largest energy shift of peak P2 (associated with the nitrogen site in the ring) should occur when crossing p*K*_a2_. Both effects are seen in the experimental data; peak P1 shifting by 1.14 eV and peak P2 shifting by 0.82 eV (compare Table 1), indicated by the light-blue dotted lines in Figure 3. P1 and P2 peak shifts which are not associated with local protonation/deprotonation are considerably smaller (0.31 eV in both cases, compare the gray dotted lines), mainly reflecting the global spectral shift. An analogous small shift of ∼0.15 eV was observed for N 1s when switching from Pro^+^ to Pro^zw^ (Figure 2A). With the same qualitative arguments, we can explain the merging of P2 and P3 for Pro^–^, giving rise to what appears as a single broad peak. As can be seen in Table 1, upon crossing p*K*_a2_, the P2 energy shift is 0.35 eV larger than that for P3. This is expected because deprotonation of NH_2_^+^ results in an increase of charge density at the N site and consequently also an increase of electron charge density near the adjacent C2 and C5 carbon atoms (associated with P3). The effect is obviously smaller for the more distant C3 and C4 atoms. Taken together, the chemical shifts occurring upon protonation are controlled by local interactions and are rather insensitive to interactions over a distance larger than a single bond. Furthermore, counterions have only a small effect on the BEs as they are largely screened by water polarization.^47^

The experimental data are directly compared with the calculated spectra in Figure 3B. The theoretical spectra of the protonated (red), zwitterionic (green), and deprotonated (blue) species have been shifted by −0.42 0.24, and 0.79 eV, respectively. This matches the spectral centroid of each spectrum with the experimental one, and compensates for an over/underestimation of the polarization screening in the model for the differently charged states. The same energy shifts have been applied to the calculated nitrogen spectra in Figure 2, shown above; i.e., the energy shifts in that figure are based on the carbon 1s comparison in Figure 3B; the as-calculated data are shown in the SI. We observe that the spectra calculated for the species Pro^+^, Pro^zw^, and Pro^–^ match well the experimental data at the respective pHs. Even the aforementioned evolution of the double-peak P2–P3 structure into a single peak is reproduced, and furthermore, the relative shifts between the higher- and lower-energy peaks are matched by the theory. This demonstrates that complex LJ-PES spectra from rather complicated aqueous-phase molecules can be reliably interpreted through comparison with theory.

The comparison with the gas-phase data is complicated by the structural changes upon solvation and the multiple conformers present in the gas phase. With the help of the nitrogen 1s PE spectra (Figure 2), we already identified the gas-phase CF1 and CF2 conformers by their distinctly different BEs. Such energy separation is, however, not observed in the gas-phase carbon 1s spectrum, and the contribution of conformers to this spectrum has not been discussed by Plekan et al. We calculated the gas-phase spectrum to investigate the spectral shape and energies associated with both conformers. The result is shown in Figure 3B, top. It is seen that the energy separation between both conformers is small; i.e., the respective spectra strongly overlap. Fitting the calculated spectra for the isomers into the measured spectra would suggest a rather large CF2:CF1 ratio, much larger than inferred from the N 1s spectra. Given the degree of overlap between the C 1s spectra of the two conformers, very small differences in the calculated BEs of each band for each individual conformer will likely significantly affect the CF2:CF1 ratio required for fitting the experimental data. This means that the conformer ratio obtained via fitting of the C 1s spectrum is much less tolerant of small differences between calculated and measured BEs than in the case of the well-separated contributions in the N 1s spectra. We thus do not place much significance on the obtained ratio here and only note that there must be some signal contribution of both conformers in the gaseous C 1s spectrum.

We briefly comment on possible orientational changes of the molecule at the surface as well as a possible change of surface propensity, as a function of charge state. This is based on the data presented, and we did not aim to systematically explore these effects here. A rough estimate of the average molecular orientation may be obtained from the relative peak areas between the isolated peak P1, originating from the carbon within the carboxylic group, and the combined signal from peaks P2 and P3, i.e., from the ring carbons. In our experiment, this sensitivity arises from a combination of interface-sensitive probing (by choice of suitable KEs) and the fact that atomic sites which are residing closer at the surface exhibit a stronger PE signal on average due to reduced inelastic electron scattering with the solvent.^48^ Assuming that variations in the photoionization cross section are negligibly small, a randomly oriented molecule should yield a ring-to-carboxylic carbon ratio of 4. For the protonated (Pro^+^) and the zwitterionic (Pro^zw^) species, we find a ratio close to that with 4.4 and 4.3, respectively. Interestingly, the ratio increases to almost 5 for deprotonated proline (Pro^–^). This seems to suggest that driven by the deprotonation of the nitrogen site, the distance of the ring to the solution surface decreases relative to the carboxylic group. We furthermore argue that protonation-state changes of proline do not lead to select noticeable surface activity, judged by the relatively high solubility, and the fact that the molecule in water always carries at least one charged group.

#### 3.1.3 Valence-Band Spectra

**Figure 4:**
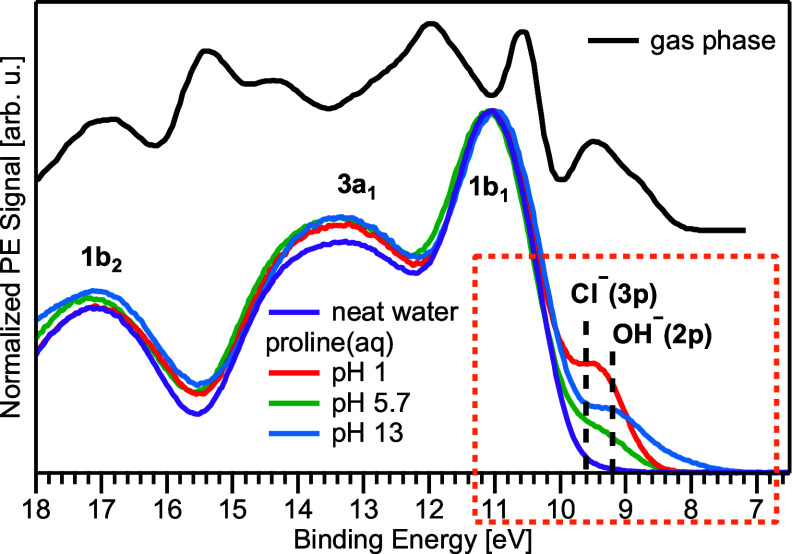
Valence PE spectra of aqueous-phase proline at pH 1 (red), 5.7 (green), and 13 (blue), measured with a photon energy of 403.08 eV (the same as used for C 1s). The purple spectrum is from neat water, where 50 mM NaCl was dissolved to maintain conductivity. Intensities are normalized to water’s 1b_1_ band. The black spectrum is from gaseous proline from ref. (16) measured with a photon energy of 99 eV. Note that the liquid-phase spectra are dominated by the solvent PE signal and are thus not directly comparable to the gas phase. HCl or NaOH was added to yield a solution with pH 1 or 13, which introduces additional Cl^–^ or OH^–^ anion signal contributions, respectively. The BEs of Cl^–^ 3p and OH^–^ 2p, 9.6 and 9.2 eV, respectively, are indicated as vertical dashed lines.^51,52^ The box (orange dotted line) indicates the energy region shown in Figure 5.

Valence-band spectra are generally less sensitive to the details of the molecular structure because of many overlapping electronic states, which is not the case for the characteristic core-level energies. Yet, they still contain important information regarding the molecular charge state. Additionally, the valence spectrum is directly related to the redox properties of the molecules.^49,50^Figure 4 displays the valence spectra of gas-phase Pro^0^ (also reproduced from ref. (16)) and aqueous-phase Pro^+^ (pH 1), Pro^zw^ (pH 5.7), and Pro^–^ (pH 13) of 1 M l-proline(aq), as well as neat liquid water for comparison. Spectra were measured from a biased liquid jet and applying the same photon energy of 403.08 eV as for the C 1s spectra; this is a considerably higher photon energy than used by Plekan et al. (99 eV) and will reflect in different ionization cross sections, although this is not relevant here. As described in the Experimental section, the bias voltage serves to drastically spread out the overlapping gas-phase water signal over a large spectral range, strongly diminishing its spectral contribution, and allows for an accurate determination of electron BEs.^22,30−32^ The liquid-phase spectra are strongly dominated by the PE features of liquid water (1b_2_, 3a_1_, and 1b_1_ photoelectrons) with the solute-specific peaks only clearly discernible in the low-BE region, occurring below the lowest BE of the (liquid) water 1b_1_ orbital (HOMO). Many solute PE features overlap with those of the solvent, especially at higher BE, but we will focus on the low-BE features here. In that same energy window, the signals from chloride (Cl^–^ 3p at 9.6 eV) and OH^–^ (O 2p at 9.2 eV)^51,52^ (from added hydrochloric acid and sodium hydroxide used for pH adjustment) contribute considerably, complicating the spectral assignment.

**Figure 5:**
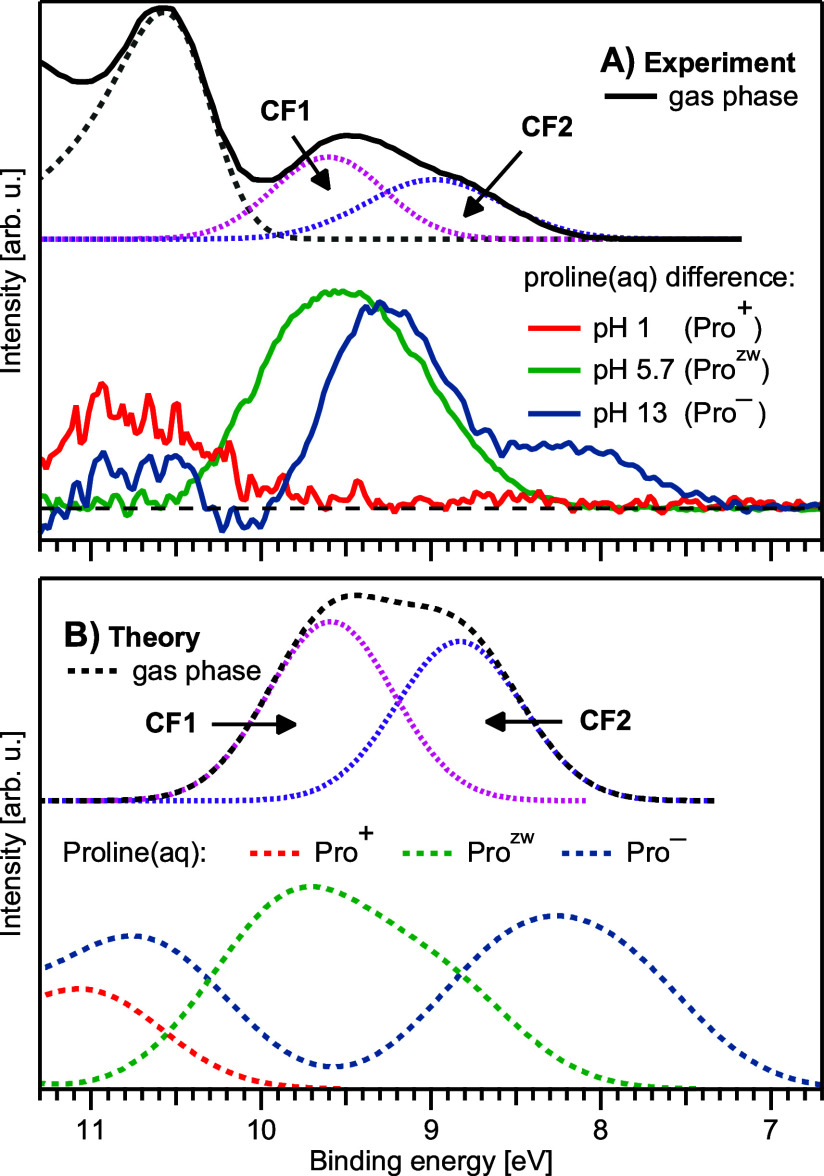
A) Valence photoelectron spectra from Figure 4 of proline(aq) at pH 1 (red), pH 5.7 (green), and pH 13 (blue) after subtraction of a reference neat-water spectrum, as well as isolated OH^–^(aq) and Cl^–^(aq) signal contributions for the pH = 1 and pH = 13 spectra, respectively, to reveal only the aqueous-proline signal contributions. We compare the results again with the gas-phase spectrum from Plekan et al. (black, top). This gas-phase spectrum has been fitted with Gaussian functions to reveal individual conformer contributions (dotted lines) in the ratio 1:1.12 as used for the N 1s spectra. B) Corresponding theoretical spectra of gaseous proline, again consisting of the two conformers CF1 and CF2 (ratio 1:1.12) as indicated, and protonated (red), zwitterionic (green), and deprotonated (blue) proline in aqueous solution; all calculated liquid-phase spectra have been uniformly shifted by 0.5 eV toward higher BE.

The low-BE energy region is shown enlarged in Figure 5, which is organized analogously to Figure 2. Figure 5A, top, displays the valence spectrum from gas-phase Pro^0^, which reveals the energetically separated CF1 and CF2 contributions, visualized by the respective Gaussian fits. In ref. (16), the features with BEs 8.95 and 9.65 eV have been assigned to the HOMO orbitals of CF1 and CF2, respectively. These findings and the positions of the peaks are also reproduced by the present calculations of Pro^0^, with its two CF1 and CF2 components (Figure 5B, top).

In Figure 5A, bottom, we present the measured valence spectra from Pro^+^, Pro^zw^, and Pro^–^, after subtraction of signal contributions from the solvent and pH-adjusting agents. The subtraction procedure was as follows. We first subtracted a scaled neat-water spectrum from the spectrum of each solution to obtain the solute signal contribution (proline plus pH-adjusting agents, where applicable). Solutions of the pH-adjusting agents (NaOH and HCl) without proline were measured separately under the same experimental conditions; subtracting a neat-water spectrum from those spectra yielded the isolated signal contribution from the pH agents. In a final step, the signal of the pH agents was subtracted from the respective solute signal, obtained as explained above. This yields the signal contribution of proline alone, which, however, entails complications, as explained in the following.

Before attempting a quantitative spectral analysis of the experimental solution spectra, we briefly comment on the expected complications. It is common practice to subtract solvent PE signal, possibly with added constituents such as HCl for lowering the pH, in the same concentration as the solution of interest, aiming at extracting the solute-only spectrum. Clearly, this is of no concern for the core-level PE spectra but often complicates analysis of valence-band spectra. One particularly complicating effect in the present case is that NaOH and HCl in solution pushes water’s autoionization equilibrium toward the production of OH^–^ and H_3_O^+^, which unavoidably alters the solvent PE spectrum. However, the associated water electronic-structure changes from these pH variations have not yet been sufficiently characterized and thus cannot be addressed in further detail here. Another concern is that bulk solution concentration may differ from the interfacial concentration. Although we employed a rather high photon energy, PES is inherently surface-sensitive and probes only the first few nanometers of a sample.

With that in mind we explore which information can and cannot be obtained starting with a more detailed description of the experimental solution spectra of Figure 5A. We will base our interpretation primarily on computed valence spectra, presented in Figure 5B; here, all aqueous-phase spectra have been shifted by 0.5 eV toward higher BE to facilitate the comparison with the experiment. Such a deviation is reasonable to expect for charged systems described within a dielectric continuum.^47^ Computed aqueous-phase valence spectra from Pro^+^, Pro^zw^, and Pro^–^ reproduce the respective experimental spectra reasonably well. The error, on the order of tenths of eVs, is typical for valence-spectra calculations based exclusively on dielectric models. For the full agreement, large-scale explicit hydration would be needed, especially for the charged species.^47^

The Pro^zw^ valence spectrum (Figure 5A, green) is the easiest to reliably extract because a distinct proline signal appears at binding energies smaller than those for water 1b_1_, and there is no signal contribution from either Cl^–^ or OH^–^ at this naturally occurring pH 5.7. This spectrum has an asymmetric shape toward smaller BEs, with a maximum near 9.6 eV, and a low-energy onset near 8.2 eV. In this case, the agreement with theory (Figure 5B, green) is excellent. Specifically, the computed spectral onset is at ∼8.0 eV, close to the experimental value of 8.2 eV. The bimodal spectral shape and the asymmetry is reproduced as well. The calculations show that the asymmetry is caused by different contributing states, with the first electron located at 8.46 eV and the two other electrons at a higher BE. Note also that the Pro^zw^ spectrum is not shifted to lower BEs with respect to gaseous Pro^0^ as one would expect for solvated species placed into a liquid phase without any attendant change in structure. This is a clear indication of chemical changes during solvation (i.e., formation of the zwitterion).

When moving from Pro^zw^ to Pro^–^, Figure 5A, blue, the valence spectrum appears to be largely shifted as a whole toward smaller BEs, the maximum now occurring near 9.2 eV, and the onset at 7.3 eV. Note again though that extracting the signal is complicated by the BE of OH^–^ (9.2 eV) which limits the reliability of the spectrum. Still, the decrease of BEs to lower values is consistent with previous studies on anions^47^ as well as with the carbon C 1s spectra. The calculated data (Figure 5B, blue) support the present assignment, with the lower-energy part of the spectrum agreeing well with the experiment. The low-energy onset is somewhat smaller, 6.8 eV, compared to 7.2 eV in the experiment, yet the shift is well reproduced. The poor agreement near ∼9.2 eV likely arises from incomplete subtraction of the OH^–^ signal.

We now turn to the Pro^+^ spectrum (Figure 5A,B, red) which is expected to be considerably blue-shifted relative to Pro^zw^ due to the stabilization of the valence electrons by the positive charge. However, experimentally, this region appears to be even less accessible for the extraction of any reliable data now due to the spectral contribution from Cl^–^ (9.6 eV) as well as the strong overlap with the solvent. An attempt to subtract that signal is presented in Figure 5A, bottom. The computed Pro^+^ spectrum indeed occurs at higher BEs, yet the apparent good match with the experiment likely misleads for the reasons given above. In conclusion, observed general trends of (onset) energy shifts reflect destabilization of the electrons by about 1 eV for each added negative charge. Our valence data may suggest to be consistent with the smaller conformer space as inferred from above core-level spectra, but additional computations and experiments would be required to support that.

Altogether we find the agreement between theory and experiment to be quite good and that the calculated spectra are of direct relevance for understanding the origins of BE shifts upon protonation/deprotonation of proline in water. Nevertheless, persistent small energetic discrepancies between theory and experiment highlight the non-negligible role of specific solute–solvent interactions.

## 4 Conclusions

We conducted core-level and valence liquid-jet photoelectron spectroscopy (LJ-PES) on l-proline in aqueous solution, supported by efficient electronic-structure calculations. The three distinct protonation states of aqueous-phase proline can be unambiguously identified through their respective N 1s, C 1s, and valence photoelectron spectra. The binding energies of N 1s and C 1s are particularly sensitive to changes in the charge states of both the amine and carboxyl groups. The N 1s spectrum is dominated by a single peak of characteristic binding energy, while the C 1s spectra exhibit three distinct peaks, corresponding to the carbon atoms of the carboxylic end, the ring carbons distant from the amine, and those near the amine, respectively. The comparison of different protonation states reveals that the spectral features are predominantly governed by the local chemical environment at the probed site. Such site sensitivity is not found in the valence spectra, due to the many overlapping valence electronic states present, further complicated by the necessity of subtracting the solvent PE signal. Nevertheless, the main trends of the ionization onsets across protonation states can be detected and are in good agreement with our calculations.

We report that the conformational space of proline is significantly reduced in the aqueous phase compared with the gas phase. In gas-phase N 1s PE spectra of proline, two peaks are clearly visible, corresponding to two dominant conformers of proline. These conformers are differentiated by the angle of the carboxylic group with respect to the heterocyclic amine. Our aqueous-phase N 1s spectra, exhibiting only a single peak, combined with our calculations of zwitterionic and neutral proline in water, demonstrate that the dominant gas-phase conformers are not present in significant populations for aqueous-phase proline. These results are consistent, albeit somewhat less pronounced due to peak overlap, in comparisons of aqueous- and gas-phase C 1s and valence-band spectra. Although the interpretation of aqueous-phase photoelectron spectra often benefits from comparison with gas-phase data, assuming that the solvent acts as a mere spectator, leading mainly to an energy shift and broadening of spectral features, our results urge caution in the case of zwitterions and when multiple low-energy conformers exist in the gas phase.

LJ-PES is becoming a recognized tool for structure determination, with potential applications even for complex biological systems, such as adenosine triphosphate and its associated Mg^2+^ complexes in aqueous solution.^23^ The technique reliably provides insight into molecular structures under extreme conditions, including those involving glucose,^41^ and even subtle hydrogen-bonding interactions, such as in indole.^53^ Thus far, the application of this technique has relied on a combination of experimental and theoretical approaches. However, for more complex systems, this approach may become impractical. Our present work shows that structural changes in biomolecules can be understood using general chemical principles, i.e., we find that the liquid-phase BEs are chiefly governed by the immediate chemical environment. It is therefore feasible to compile a table of BEs depending on their chemical neighbors and solvents. Such tables are routinely used in solid-state PES, although referenced to the Fermi level and not the vacuum level, as done here. With this approach, many chemically relevant questions could be answered, even for more complex systems. We are, for example, able to clearly identify the central carbon atoms involved in the peptide bond. A system formed by further functionalization with, e.g., a long alkyl chain will not obscure these findings. Further studies are needed before we can completely rely on the accumulated experimental data in the still relatively young field of LJ-PES.

## Data Availability

The data of relevance to this study have been deposited at the following DOI: 10.5281/zenodo.13349483.

## Supporting Information Available

Comparison with a hypothetical neutral proline in aqueous solution; Subtraction of a small signal contribution of Pro^zw^ in the Pro^+^ PE spectrum; Unshifted theoretical core spectra; Comparison between the experimental data of two measurement campaigns; 3D representation of proline structures used for the calculations; Calculated binding energies of proline embedded in a dielectric continuum; Energies of different proline conformers; Cartesian coordinates of the optimized molecules (PDF)

## References

[ref1] Morris A. L.; MacArthur M. W.; Hutchinson E. G.; Thornton J. M. Stereochemical quality of protein structure coordinates. Proteins 1992, 12, 345–364. 10.1002/prot.340120407. 1579569

[ref2] Karna E.; Szoka L.; Huynh T. Y. L.; Palka J. A. Proline-dependent regulation of collagen metabolism. Cell. Mol. Life Sci. 2020, 77, 1911–1918. 10.1007/s00018-019-03363-3. 31740988 PMC7228914

[ref3] Patriarca E. J.; Cermola F.; D’Aniello C.; Fico A.; Guardiola O.; De Cesare D.; Minchiotti G. The multifaceted roles of proline in cell behavior. Front. Cell Dev. Biol. 2021, 9, 728576 10.3389/fcell.2021.728576. 34458276 PMC8397452

[ref4] Kavi Kishor P. B.; Suravajhala P.; Rathnagiri P.; Sreenivasulu N. Intriguing role of proline in redox potential conferring high temperature stress tolerance. Front. Plant Sci. 2022, 13, 867531 10.3389/fpls.2022.867531. 35795343 PMC9252438

[ref5] Liang X.; Zhang L.; Natarajan S. K.; Becker D. F. Proline mechanisms of stress survival. Antioxid. Redox Signaling 2013, 19, 998–1011. 10.1089/ars.2012.5074. PMC3763223 23581681

[ref6] Li P.; Wu G. Roles of dietary glycine, proline, and hydroxyproline in collagen synthesis and animal growth. Amino Acids 2018, 50, 29–38. 10.1007/s00726-017-2490-6. 28929384

[ref7] Yang G.; Zhou L.; Chen Y. Stabilization of zwitterionic versus canonical proline by water molecules. SpringerPlus 2016, 5, 19 10.1186/s40064-015-1661-8. 26759758 PMC4703596

[ref8] Levy Y.; Onuchic J. N. Water Mediation in Protein Folding and Molecular Recognition. Annu. Rev. Biophys. Biomol. Struct. 2006, 35, 389–415. 10.1146/annurev.biophys.35.040405.102134. 16689642

[ref9] Biedermannová L.; Schneider B. Hydration of proteins and nucleic acids: Advances in experiment and theory. A review. Biochim. Biophys. Acta, Gen. Subj. 2016, 1860, 1821–1835. 10.1016/j.bbagen.2016.05.036. 27241846

[ref10] Vishveshwara S.; Pople J. A. Molecular orbital theory of the electronic structures of organic compounds. 32. Conformations of glycine and related systems. J. Am. Chem. Soc. 1977, 99, 2422–2426. 10.1021/ja00450a004.

[ref11] Ottosson N.; Børve K. J.; Spångberg D.; Bergersen H.; Sæthre L. J.; Faubel M.; Pokapanich W.; Öhrwall G.; Björneholm O.; Winter B. On The Origins of Core-Electron Chemical Shifts of Small Biomolecules in Aqueous Solution: Insights from Photoemission and Ab Initio Calculations of Glycine_aq_. J. Am. Chem. Soc. 2011, 133, 3120–3130. 10.1021/ja110321q. 21319819

[ref12] Belyakov A. V.; Gureev M. A.; Garabadzhiu A. V.; Losev V. A.; Rykov A. N. Determination of the molecular structure of gaseous proline by electron diffraction, supported by microwave and quantum chemical data. Struct. Chem. 2015, 26, 1489–1500. 10.1007/s11224-015-0589-5.

[ref13] Kim T.-Y.; Valentine S. J.; Clemmer D. E.; Reilly J. P. Gas-phase conformation-specific photofragmentation of proline-containing peptide ions. J. Am. Soc. Mass Spectrom. 2010, 21, 1455–1465. 10.1016/j.jasms.2010.04.007. 20483641

[ref14] Stepanian S. G.; Reva I. D.; Radchenko E. D.; Adamowicz L. Conformers of Nonionized Proline. Matrix-Isolation Infrared and Post-Hartree–Fock ab Initio Study. J. Phys. Chem. A 2001, 105, 10664–10672. 10.1021/jp011708i.

[ref15] Dehareng D.; Dive G. Vertical Ionization Energies of α-L-Amino Acids as a Function of Their Conformation: an Ab Initio Study. Int. J. Mol. Sci. 2004, 5, 301–332. 10.3390/i5110301.

[ref16] Plekan O.; Feyer V.; Richter R.; Coreno M.; de Simone M.; Prince K. C.; Carravetta V. Investigation of the Amino Acids Glycine, Proline, and Methionine by Photoemission Spectroscopy. J. Phys. Chem. A 2007, 111, 10998–11005. 10.1021/jp075384v. 17918919

[ref17] Plekan O.; Feyer V.; Richter R.; Coreno M.; de Simone M.; Prince K. C.; Carravetta V. Photoemission and the shape of amino acids. Chem. Phys. Lett. 2007, 442, 429–433. 10.1016/j.cplett.2007.05.110. 17918919

[ref18] Czinki E.; Császár A. G. Conformers of Gaseous Proline. Chem. Eur. J. 2003, 9, 1008–1019. 10.1002/chem.200390103. 12584718

[ref19] Smith P. K.; Gorham A. T.; Smith E. R. B. Substances: VII. The Ionization of some Hydroxyamino Acids and Proline in Aqueous Solution from One to Fifty Degrees. J. Biol. Chem. 1942, 144, 737–745. 10.1016/S0021-9258(18)72499-X.

[ref20] Meyer F.; Hauschild D.; Benkert A.; Blum M.; Yang W.; Reinert F.; Heske C.; Zharnikov M.; Weinhardt L. Resonant inelastic soft X-ray scattering and X-ray emission spectroscopy of solid proline and proline solutions. J. Phys. Chem. B 2022, 126, 10185–10193. 10.1021/acs.jpcb.2c06557. 36418225 PMC9744097

[ref21] Messer B. M.; Cappa C. D.; Smith J. D.; Drisdell W. S.; Schwartz C. P.; Cohen R. C.; Saykally R. J. Local hydration environments of amino acids and dipeptides studied by X-ray spectroscopy of liquid microjets. J. Phys. Chem. B 2005, 109, 21640–21646. 10.1021/jp053802v. 16853810

[ref22] Winter B.; Thürmer S.; Wilkinson I. Absolute Electronic Energetics and Quantitative Work Functions of Liquids from Photoelectron Spectroscopy. Acc. Chem. Res. 2023, 56, 77–85. 10.1021/acs.accounts.2c00548. 36599420 PMC9850918

[ref23] Mudryk K.; Lee C.; Tomaník L.; Malerz S.; Trinter F.; Hergenhahn U.; Neumark D. M.; Slavíček P.; Bradforth S.; Winter B. How Does Mg^2+^_(aq)_ Interact with ATP_(aq)_? Biomolecular Structure through the Lens of Liquid-Jet Photoemission Spectroscopy. J. Am. Chem. Soc. 2024, 146, 16062–16075. 10.1021/jacs.4c03174. 38802319 PMC11177255

[ref24] Tomaník L.; Pugini M.; Mudryk K.; Thürmer S.; Stemer D.; Credidio B.; Trinter F.; Winter B.; Slavíček P. Liquid-jet photoemission spectroscopy as a structural tool: site-specific acid–base chemistry of vitamin C. Phys. Chem. Chem. Phys. 2024, 26, 19673–19684. 10.1039/D4CP01521E. 38963770 PMC11267885

[ref25] Winter B. Liquid microjet for photoelectron spectroscopy. Nucl. Instrum. Methods Phys. Res., Sect. A 2009, 601, 139–150. 10.1016/j.nima.2008.12.108.

[ref26] Viefhaus J.; Scholz F.; Deinert S.; Glaser L.; Ilchen M.; Seltmann J.; Walter P.; Siewert F. The Variable Polarization XUV Beamline P04 at PETRA III: Optics, mechanics and their performance. Nucl. Instrum. Methods Phys. Res., Sect. A 2013, 710, 151–154. 10.1016/j.nima.2012.10.110.

[ref27] Malerz S.; Haak H.; Trinter F.; Stephansen A. B.; Kolbeck C.; Pohl M.; Hergenhahn U.; Meijer G.; Winter B. A setup for studies of photoelectron circular dichroism from chiral molecules in aqueous solution. Rev. Sci. Instrum. 2022, 93, 015101 10.1063/5.0072346. 35104975

[ref28] Buck J.; Bagschik K.; Glaser L.; Scholz F.; Seltmann J.; Viefhaus J. Progress report on the XUV online diagnostic unit for the highly accurate determination of SR properties. AIP Conf. Proc. 2019, 2054, 060057 10.1063/1.5084688.

[ref29] The Merck Index: An Encyclopedia of Chemicals, Drugs, and Biologicals; Royal Society of Chemistry, 2013.

[ref30] Thürmer S.; Malerz S.; Trinter F.; Hergenhahn U.; Lee C.; Neumark D. M.; Meijer G.; Winter B.; Wilkinson I. Accurate Vertical Ionization Energy and Work Function Determinations of Liquid Water and Aqueous Solutions. Chem. Sci. 2021, 12, 10558–10582. 10.1039/D1SC01908B. 34447550 PMC8356740

[ref31] Credidio B.; Pugini M.; Malerz S.; Trinter F.; Hergenhahn U.; Wilkinson I.; Thürmer S.; Winter B. Quantitative electronic structure and work-function changes of liquid water induced by solute. Phys. Chem. Chem. Phys. 2022, 24, 1310–1325. 10.1039/D1CP03165A. 34604895 PMC8768487

[ref32] Pugini M.; Credidio B.; Walter I.; Malerz S.; Trinter F.; Stemer D.; Hergenhahn U.; Meijer G.; Wilkinson I.; Winter B.; Thürmer S. How to measure work functions from aqueous solutions. Chem. Sci. 2023, 14, 9574–9588. 10.1039/D3SC01740K. 37712029 PMC10498509

[ref33] Saha A.; Mahali K.; Ganai S.; Mukherjee P.; Shrestha N. K.; Henaish A. M. A.; Ahmed J.; Kundu S.; Roy S. Solubility and the solution thermodynamics of l-proline in the aqueous binary mixture of NaCl and KCl solution. J. Mol. Liq. 2023, 391, 123352 10.1016/j.molliq.2023.123352.

[ref34] Chai J.-D.; Head-Gordon M. Long-range corrected hybrid density functionals with damped atom-atom dispersion corrections. Phys. Chem. Chem. Phys. 2008, 10, 6615–6620. 10.1039/b810189b. 18989472

[ref35] Mennucci B.; Tomasi J. Continuum solvation models: A new approach to the problem of solute’s charge distribution and cavity boundaries. J. Chem. Phys. 1997, 106, 5151–5158. 10.1063/1.473558.

[ref36] Cancès E.; Mennucci B.; Tomasi J. A new integral equation formalism for the polarizable continuum model: Theoretical background and applications to isotropic and anisotropic dielectrics. J. Chem. Phys. 1997, 107, 3032–3041. 10.1063/1.474659.

[ref37] Gilbert A. T. B.; Besley N. A.; Gill P. M. W. Self-consistent field calculations of excited states using the maximum overlap method (MOM). J. Phys. Chem. A 2008, 112, 13164–13171. 10.1021/jp801738f. 18729344

[ref38] Yanai T.; Tew D. P.; Handy N. C. A new hybrid exchange–correlation functional using the Coulomb-attenuating method (CAM-B3LYP). Chem. Phys. Lett. 2004, 393, 51–57. 10.1016/j.cplett.2004.06.011.

[ref39] Dunning T. H. Jr Gaussian basis sets for use in correlated molecular calculations. I. The atoms boron through neon and hydrogen. J. Chem. Phys. 1989, 90, 1007–1023. 10.1063/1.456153.

[ref40] Kendall R. A.; Dunning T. H. Jr; Harrison R. J. Electron affinities of the first-row atoms revisited. Systematic basis sets and wave functions. J. Chem. Phys. 1992, 96, 6796–6806. 10.1063/1.462569.

[ref41] Malerz S.; Mudryk K.; Tomaník L.; Stemer D.; Hergenhahn U.; Buttersack T.; Trinter F.; Seidel R.; Quevedo W.; Goy C.; Wilkinson I.; Thürmer S.; Slavíček P.; Winter B. Following in Emil Fischer’s Footsteps: A Site-Selective Probe of Glucose Acid–Base Chemistry. J. Phys. Chem. A 2021, 125, 6881–6892. 10.1021/acs.jpca.1c04695. 34328745 PMC8381351

[ref42] Pluhařová E.; Slavíček P.; Jungwirth P. Modeling Photoionization of Aqueous DNA and Its Components. Acc. Chem. Res. 2015, 48, 1209–1217. 10.1021/ar500366z. 25738773

[ref43] Jagoda-Cwiklik B.; Slavícek P.; Cwiklik L.; Nolting D.; Winter B.; Jungwirth P. Ionization of imidazole in the gas phase, microhydrated environments, and in aqueous solution. J. Phys. Chem. A 2008, 112, 3499–3505. 10.1021/jp711476g. 18335914

[ref44] Epifanovsky E.; Gilbert A. T. B.; Feng X.; Lee J.; Mao Y.; Mardirossian N.; Pokhilko P.; White A. F.; Coons M. P.; Dempwolff A. L.; Gan Z.; et al. Software for the frontiers of quantum chemistry: An overview of developments in the Q-Chem 5 package. J. Chem. Phys. 2021, 155, 084801. 34470363 10.1063/5.0055522 PMC9984241

[ref45] Frisch M. J.; Trucks G. W.; Schlegel H. B.; Scuseria G. E.; Robb M. A.; Cheeseman J. R.; Scalmani G.; Barone V.; Mennucci B.; Petersson G. A., Gaussian 09, Revision D.01. Gaussian, Inc.: Wallingford, CT, 2009.

[ref46] Suzuki T. Ultrafast photoelectron spectroscopy of aqueous solutions. J. Chem. Phys. 2019, 151, 090901 10.1063/1.5098402. 31492071

[ref47] Pluhařová E.; Ončák M.; Seidel R.; Schroeder C.; Schroeder W.; Winter B.; Bradforth S. E.; Jungwirth P.; Slavíček P. Transforming Anion Instability into Stability: Contrasting Photoionization of Three Protonation Forms of the Phosphate Ion Upon Moving into Water. J. Phys. Chem. B 2012, 116, 13254–13264. 10.1021/jp306348b. 22970895

[ref48] Björneholm O.; Öhrwall G.; Naves de Brito A.; Ågren H.; Carravetta V. Superficial Tale of Two Functional Groups: On the Surface Propensity of Aqueous Carboxylic Acids, Alkyl Amines, and Amino Acids. Acc. Chem. Res. 2022, 55, 3285–3293. 10.1021/acs.accounts.2c00494. 36472092 PMC9730837

[ref49] Slavíček P.; Winter B.; Faubel M.; Bradforth S. E.; Jungwirth P. Ionization Energies of Aqueous Nucleic Acids: Photoelectron Spectroscopy of Pyrimidine Nucleosides and ab Initio Calculations. J. Am. Chem. Soc. 2009, 131, 6460–6467. 10.1021/ja8091246. 19374336

[ref50] Schroeder C. A.; Pluhařová E.; Seidel R.; Schroeder W. P.; Faubel M.; Slavíček P.; Winter B.; Jungwirth P.; Bradforth S. E. Oxidation Half-Reaction of Aqueous Nucleosides and Nucleotides via Photoelectron Spectroscopy Augmented by ab Initio Calculations. J. Am. Chem. Soc. 2015, 137, 201–209. 10.1021/ja508149e. 25551179

[ref51] Winter B.; Faubel M.; Hertel I. V.; Pettenkofer C.; Bradforth S. E.; Jagoda-Cwiklik B.; Cwiklik L.; Jungwirth P. Electron Binding Energies of Hydrated H_3_O^+^ and OH^-^: Photoelectron Spectroscopy of Aqueous Acid and Base Solutions Combined with Electronic Structure Calculations. J. Am. Chem. Soc. 2006, 128, 3864–3865. 10.1021/ja0579154. 16551066

[ref52] Winter B.; Weber R.; Hertel I. V.; Faubel M.; Jungwirth P.; Brown E. C.; Bradforth S. E. Electron binding energies of aqueous alkali and halide ions: EUV photoelectron spectroscopy of liquid solutions and combined ab initio and molecular dynamics calculations. J. Am. Chem. Soc. 2005, 127, 7203–7214. 10.1021/ja042908l. 15884962

[ref53] He L.; Tomaník L.; Malerz S.; Trinter F.; Trippel S.; Belina M.; Slavíček P.; Winter B.; Küpper J. Specific versus Nonspecific Solvent Interactions of a Biomolecule in Water. J. Phys. Chem. Lett. 2023, 14, 10499–10508. 10.1021/acs.jpclett.3c01763. 37970807 PMC10683073

