# Supplementary material for: From Gas to Solution: The Changing Neutral Structure of Proline upon Solvation
Source: J Phys Chem A. 2024 Nov 13;128(47):10202–12. doi: 10.1021/acs.jpca.4c05628 (PMC11613541; doi:10.1021/acs.jpca.4c05628)
Supplement: Supplementary file 1 — jp4c05628_si_001.pdf [file jp4c05628_si_001.pdf]

Supporting Information

From Gas to Solution: The Changing Neutral Structure of Proline Upon Solvation

Bruno Credidio, Stephan Thürmer, Dominik Stemer, Michele Pugini, Florian Trinter, Jakub Vokrouhlický, Petr Slavíček, and Bernd Winter

Comparison with a hypothetical neutral proline in aqueous solution

The calculated N 1s and C 1s PE spectra of the hypothetical neutral proline conformers are shown in Figure S1, panels A and B, respectively, and are each compared to the spectra of Pro^{zw} in aqueous solution (the real neutral species in solution). We furthermore add the gas-phase spectra from Ref. [17] after a applying a shift of 1.0 eV towards lower binding energies (BEs) to simulate a gas-liquid shift from screening by the solvent; this gas-liquid shift has been identified by analyzing the energetic difference in the spectra centroid between the gas-phase neutral and the aqueous-phase zwitterionic species in the main text. Conformers CF1 and CF2 are shown as a purple and pink dashed line, respectively. All theoretical spectra have been energetically shifted by 0.24 eV towards higher BEs, analogous to the shift applied to the one of the zwitterion in Figures 2 and 3 of the main text; this shift was argued to represent a compensation for the solvent screening which could not be fully captured in the theory. The conformer intensities are scaled to match the 1:1.12 ratio (N 1s) and 1:4 ratio (C 1s) as discussed in the main text; the sum is shown in brown. We remind the reader that the nitrogen site of the zwitterion is doubly protonated, while all other species shown here are singly protonated.

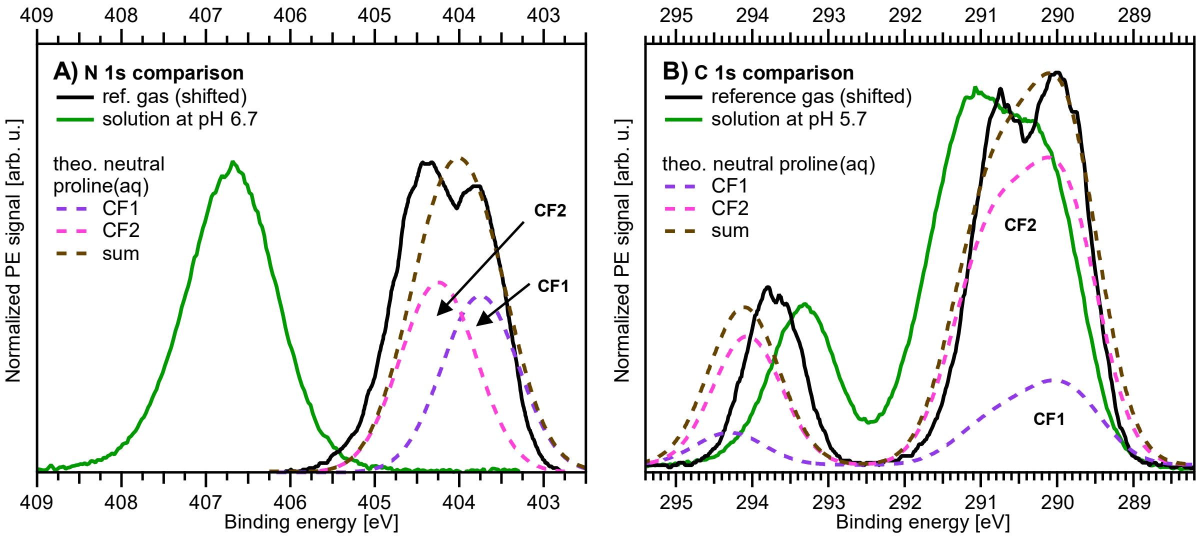

Figure S1: Calculated PE spectra of a hypothetical neutral proline(aq) as two conformers CF1 and CF2, which are compared to the gas phase from Ref. [17]: **A)** N 1s and **B)** C 1s spectra. The neutral conformers are added to yield the sum in brown. The gas phase has been shifted by 1.0 eV towards lower BE to simulate the gas-liquid shift. A 0.24 eV shift towards higher BEs has been applied to the theory.

It becomes clear that Pro^{zw} and Pro^{0} in solution are different in multiple ways: The N 1s peaks (Figure S1A) have significantly lower BE for Pro^{0} than Pro^{zw}, which is expected due to the different protonation states of nitrogen; note that a shift of 2.7 eV has been associated with the protonation of this site. The peak position of Pro^{0}(aq) matches quite well the position of Pro^{0}(gas) after the solvation shift is applied, yet the peak separation is reduced for the former due to the screening of the hydrogen bond interaction by water, as discussed in the main text. For the C 1s spectra (Figure S1B), peak positions match well for Pro^{0} in the gas and liquid phase, but are significantly offset compared to Pro^{zw}. The peak corresponding to the carboxylic group has higher binding energy in Pro^{0} than in Pro^{zw}, while the opposite is true for the ring carbons. This is the expected behavior when comparing the electron densities of Pro^{0} and Pro^{zw}: Pro^{zw} is negatively charged at the oxygen of the carboxylic group, which increases the electronic density at the carbon (reduced BE), while the ring carbons are affected by a positively charged nitrogen-atom site (increased BE). This comparison confirms that the zwitterion is distinctly different from a (hypothetical) solvated neutral species, and aqueous-phase properties cannot be accurately represented by a neutral gas phase.

Subtraction of a small signal contribution of Pro^{zw} in the Pro^{+} PE spectrum

The measured proline solution at pH = 1 is expected to contain roughly 10% of zwitterions due to the close pH value to proline's pK_{a1} of 1.95. To compensate, we subtracted a fraction of the signal of the pH 5.7 solution from the pH 1.0 solution. This is demonstrated in Figure S2 (panels A and B, respectively) for both the N 1s and C 1s spectra. The scaling factor was chosen such that the area under the pH = 5.7 curve matched 10% of the area under the one for pH = 1.0. The resulting difference spectrum, red line, is the one used as the one for the deprotonated species in Figures 2 and 3 of the main text, respectively.

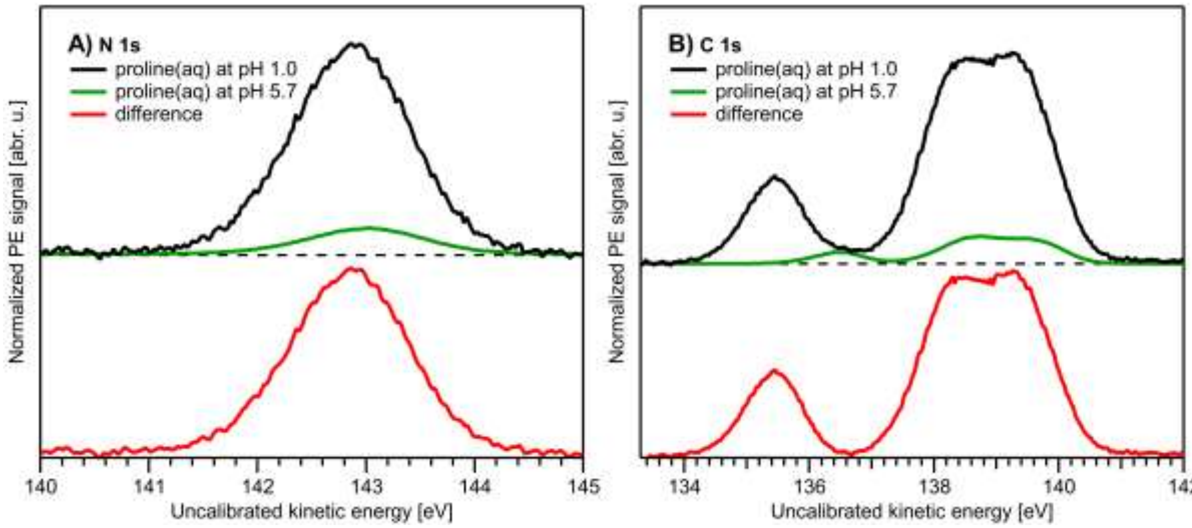

Figure S2: Demonstration of the subtraction method to compensate for 10% of zwitterions to the N 1s **A)** and C 1s **B)** PE spectrum of the pH=1 solution. In each case, the black line is the as-measured PE spectrum of the pH = 1.0 solution, where the signal of the pH = 5.7 solution (green) is subtracted after scaling to match the area ratio.

Unshifted theoretical core spectra

Figure S3 displays the theoretical N 1s and C 1s spectra of aqueous proline as is, *i.e.*, without energy shifts applied. The same data was shown shifted in Figures 2B and 3B, where the values for these energy shifts were determined by comparing the spectral centroid of experimental and theoretical C 1s spectra. This was done for compensation of an over-/under-estimation of the polarization screening in the model.

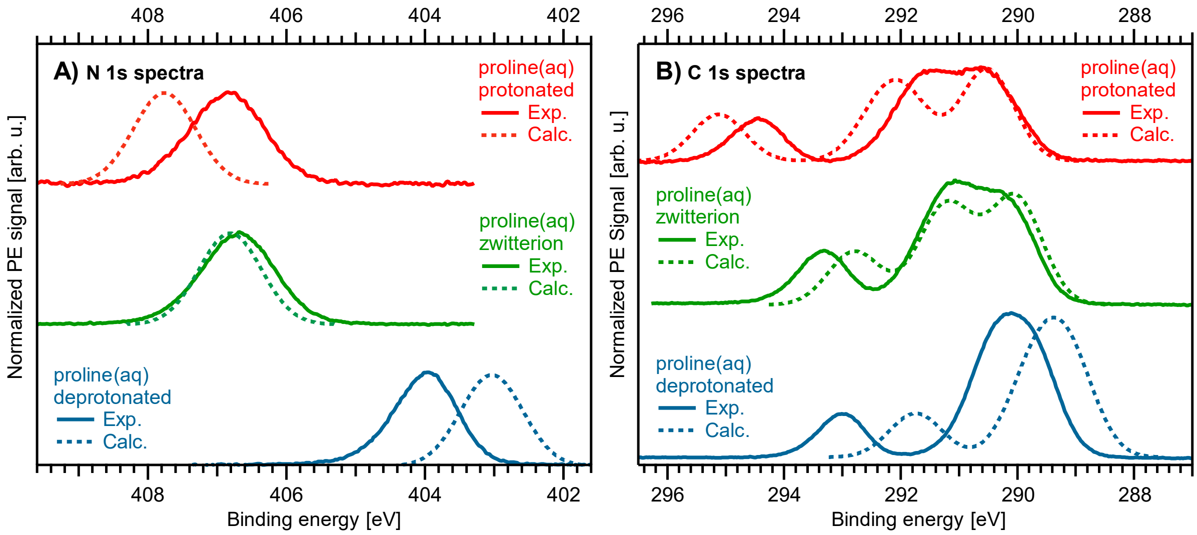

Figure S3: A) N 1s and **B)** C 1s theoretical spectra without the energy shift applied in Figures 2B and 3B of the main text.

Comparison between the experimental data of two measurement campaigns

C 1s PE spectra from 1 M L-proline were recorded in two measurement campaigns: The first set was measured in December 2022 at a photon energy of 379.66 ± 0.08 eV. A repeat measurement was done in March 2023 at a photon energy of 403.08 ± 0.03 eV. Figure S4 compares the PE spectra of both campaigns, which yield essentially identical results. The pH value to produce the zwitterionic species in solution was slightly different: In the first campaign the pH value was adjusted to 6.7, while being left unadjusted (yielding a value of 5.7) in the second campaign. Both pH values are sufficiently far away from both pK_a values, which ensures that the zwitterion is the only species in either case. Also, the pH = 1 solution in the second campaign showed a higher signal contribution of the zwitterion of about 45%, which indicates that the real pH was likely closer to the pKa_1 value of 1.95. In Figure S4, the signal contributions of the zwitterion are subtracted from the pH 1 PE spectra in both cases, which ultimately yields an identical result. Still, for Figure 3 of the main text the result of the 2022 campaign was used.

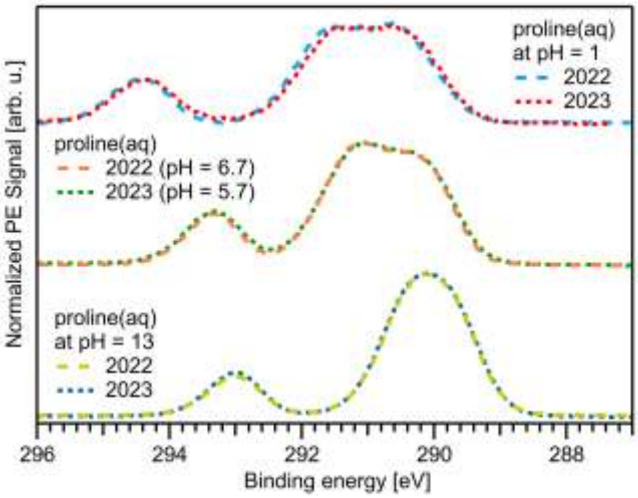

Figure S4: Comparison of the experimental C 1s PE spectra from 1 M L-proline(aq) measured in two measurement campaigns. **Top:** pH = 1 solution (light-blue dashed curve = 2022, red dotted curve = 2023). **Center:** Solutions at intermediate pH. The pH value for the 2022 campaign (orange dashed) was adjusted to yield a pH close to 7, while the pH value was not adjusted in the campaign in 2023 and yielded a natural pH of 5.7. **Bottom:** Solutions at pH = 13 (yellow dashed = 2022, blue dotted = 2023).

3D representation of proline structures used for the calculations

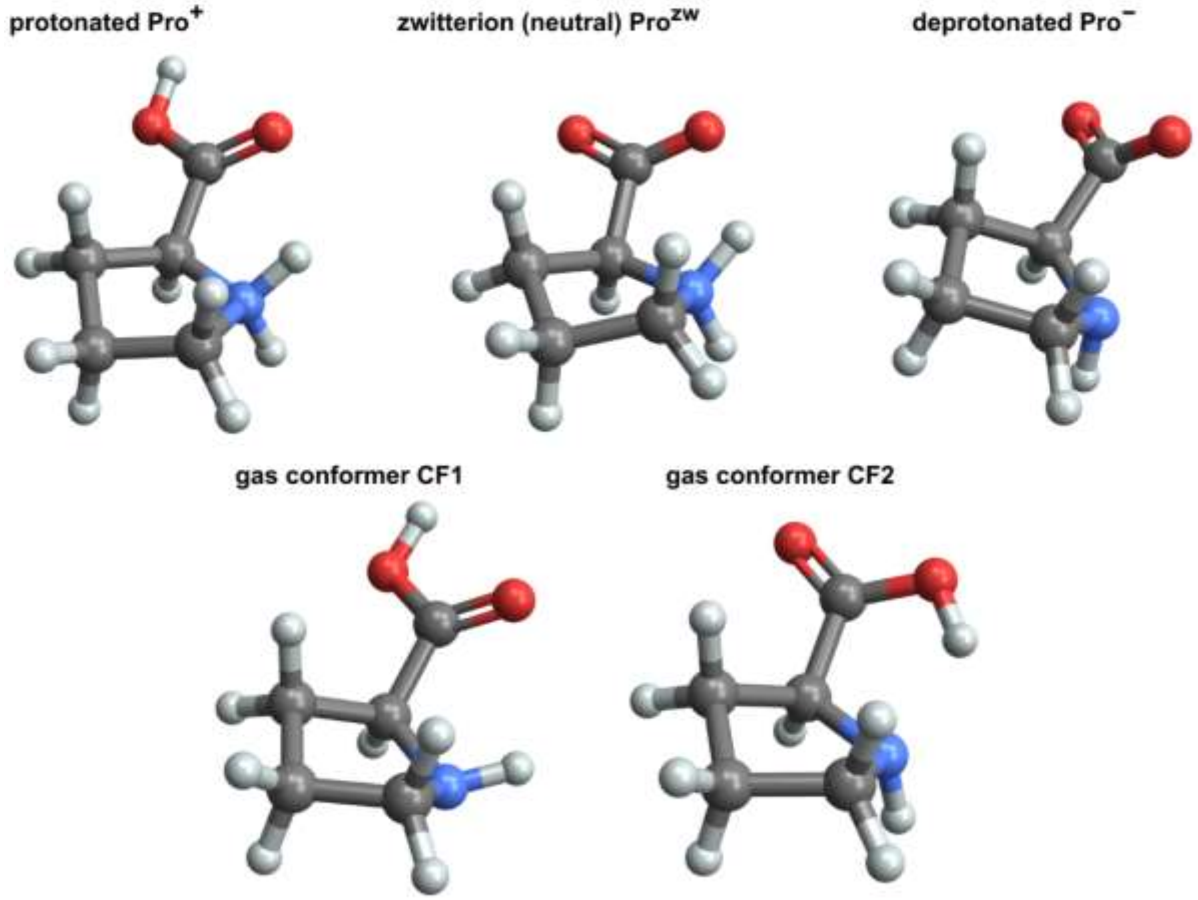

Figure S5: 3D representation of the structures used for calculating the PE spectra. **Top:** Protonated, zwitterionic, and deprotonated proline embedded in a dielectric continuum. **Bottom:** Proline conformers CF1 and CF2 in the gas phase.

Calculated binding energies of proline embedded in a dielectric continuum

Table 1: N 1s binding energies [eV] for proline.

Proline form	Neutral	Protonated	Deprotonated	Zwitterion
**BE [eV]**	403.5096	407.7601	403.0335	406.8104

Table 2: C 1s binding energies [eV] for proline.

Carbon atom	Neutral	Protonated	Deprotonated	Zwitterion
**C1**	294.0546	295.1230	291.7416	292.7919
**C2**	290.7157	292.3322	289.5756	291.3688
**C3**	290.1361	291.8559	289.8177	291.0613
**C4**	289.6735	290.5960	289.0858	290.0548
**C5**	289.4858	290.4409	289.1184	289.9919

Table 3: Lowest valence energies [eV] for proline.

Proline form	Neutral	Protonated	Deprotonated	Zwitterion
**VE [eV]**	8.0083	10.5852	7.3444	8.4573

Energies of different proline conformers

Table 4: N 1s binding energies [eV] of proline conformers CF1 and CF2, embedded in a dielectric continuum and in the gas phase.

Conformer	CF1 (dielec.)	CF2 (dielec.)	CF1 (gas)	CF2 (gas)
**BE [eV]**	403.5096	404.0077	404.3886	405.3030

Table 5: C 1s binding energies [eV] of proline conformers CF1 and CF2, embedded in a dielectric continuum and in the gas phase.

Carbon atom	CF1 (dielec.)	CF2 (dielec.)	CF1 (gas)	CF2 (gas)
**C1**	294.0546	293.8233	294.9253	294.5008
**C2**	290.7157	290.8191	291.6736	291.9049
**C3**	290.1361	290.3865	291.1810	291.8368
**C4**	289.6735	289.7443	290.7701	290.9606
**C5**	289.4858	289.5701	290.6069	290.9960

Table 6: Valence energies [eV] of the HOMO electron of proline conformers CF1 and CF2, embedded in a dielectric continuum and in the gas phase.

Conformer	CF1 (dielec.)	CF2 (dielec.)	CF1 (gas)	CF2 (gas)
**VE [eV]**	8.0056	8.5961	8.8328	9.5893

Cartesian coordinates of the optimized molecules

conformer CF1, neutral molecule, optimization in the dielectric continuum

C	1.1414077429	0.6506300782	1.8031538620
C	1.9702599436	0.9476756404	0.5450027285
C	0.9588033142	0.7645010096	-0.6265400837
N	-0.2632931635	0.2510842684	-0.0132186473
C	0.0932669024	-0.3298964967	1.2868281333
C	1.5301783209	-0.1613577773	-1.6856481325
O	1.1228261088	-1.2756503522	-1.9446236063
O	2.5644989271	0.3995726700	-2.3275708818
H	2.7909801993	0.2281874139	0.4570293887
H	1.7477320901	0.2364805896	2.6129214054
H	-0.7911497025	-0.3950179106	1.9270535554
H	-0.7196734555	-0.4257576992	-0.6172324402
H	2.9037303612	-0.2310057923	-2.9850650504
H	0.6540669473	1.5605168981	2.1695976599
H	2.4101147488	1.9468631552	0.5416834967
H	0.5304565203	-1.3377629749	1.1954791500
H	0.7658151945	1.7232452799	-1.1220265376

conformer CF1, neutral molecule, optimization in the gas phase

C	2.1012865840	0.6959209680	-0.2667768338
C	0.6798163163	1.2620973664	-0.1488620758
C	-0.0867512494	0.1705517714	0.6531191002
N	0.8117795326	-0.9757777154	0.7167298984
C	1.8501764762	-0.8088064206	-0.3023116055
C	-1.4149263630	-0.1538247937	-0.0054506216

O	-1.7086273540	-1.2082196539	-0.5216756444
O	-2.2683780030	0.8869009788	0.0490595602
H	0.2413214052	1.3798826497	-1.1459977685
H	2.6255314763	1.0641980227	-1.1530989942
H	2.7339286398	-1.3987613880	-0.0412037686
H	0.3029653761	-1.8472161503	0.6070996336
H	-3.0903480574	0.6106498402	-0.3877895541
H	2.6944366486	0.9516007141	0.6178238765
H	0.6347771373	2.2349126830	0.3451311350
H	1.5146816998	-1.1139212826	-1.3082661363
H	-0.3184819451	0.5301191794	1.6637775230

conformer CF2, neutral molecule, optimization in the dielectric continuum

N	0.3399837898	-0.5542325685	1.6413876408
C	1.2347056270	0.5973875537	1.8839591593
C	1.8590184950	0.9093464054	0.5230067765
C	2.0570009767	-0.4890473803	-0.0695605155
C	0.8173915832	-1.2655047025	0.4406286172
C	1.1573840021	-2.7093786939	0.8053071813
O	0.9838486561	-2.9906352554	2.0912211316
O	1.5600296858	-3.5324277745	0.0059134303
H	2.9769945630	-0.9362675883	0.3235487090
H	2.7954924234	1.4687503573	0.5996420612
H	0.6702593914	1.4270137934	2.3151101401
H	-0.6171832940	-0.2446218163	1.5145136174
H	1.1589556817	1.4898847887	-0.0890706671
H	2.1181948875	-0.5003160321	-1.1594998412
H	2.0096256836	0.3050374396	2.6024379780
H	0.0404747097	-1.3025158559	-0.3292730598
H	0.6347791377	-2.1349906703	2.4736186409

conformer CF2, neutral molecule, optimization in the gas phase

N	-0.5620686344	1.0884376698	0.4378332171
C	-1.7387965736	0.8669455048	-0.4229188044
C	-2.0802283941	-0.6157551546	-0.2585790637
C	-0.6908375722	-1.2589207091	-0.1842516561
C	0.1664028133	-0.1921597117	0.5449247662
C	1.5659255720	-0.0697336111	-0.0700029081
O	1.8510102327	1.1479988637	-0.5326477749
O	2.3333935161	-0.9994080635	-0.1326169899
H	-0.2962996100	-1.4353765690	-1.1907396384
H	-2.6868429579	-1.0104019363	-1.0790174667
H	-2.5449132109	1.5501864088	-0.1436206366
H	-0.8481046621	1.4146292497	1.3539597518
H	-2.6302878287	-0.7710990925	0.6780454313
H	-0.6764919256	-2.2151332591	0.3419251197
H	-1.4647794194	1.0820493884	-1.4634222614
H	0.3115923955	-0.4630885539	1.5959764168
H	1.0491546017	1.6896904291	-0.3350761118

deprotonated structure, optimization in the dielectric continuum

N	1.1395088525	0.4261399073	1.9775782090
C	1.6602622610	1.0218066537	0.7389632446
C	1.3433769535	0.0049319856	-0.3619422019
C	1.6140147761	-1.3256034855	0.3506410333
C	1.2715917573	-1.0405666629	1.8480194939
C	2.3452829202	-1.6822406688	2.7539652905
O	3.3362436030	-0.9943030363	3.1085880752
O	2.1561053423	-2.9033743685	3.0242479825
H	2.6735091949	-1.5906214158	0.2482389282
H	1.9498988362	0.1352378014	-1.2642126598
H	1.2138033157	2.0076911437	0.5768100822
H	0.2859998053	0.0854472202	-0.6446957312
H	1.0271746083	-2.1568621494	-0.0495271263
H	2.7459680251	1.1542401847	0.8298435998
H	0.3138019492	-1.5028612137	2.1070402859
H	0.1486297995	0.6442521044	2.0331164941

protonated structure, optimization in the dielectric continuum

N	0.0650642051	-0.3291884075	1.7011146282
C	1.2984542114	0.5018788557	1.9838521979
C	1.6527241454	1.0793789589	0.6209126371
C	1.3532732685	-0.0794913656	-0.3324080455
C	0.0463771531	-0.6682148769	0.2322365992
C	-0.0759360417	-2.1728503347	0.0956048556
O	-0.0772049688	-2.9282250230	1.0436215627
O	-0.1575054688	-2.5355468207	-1.1743762343
H	2.1564281682	-0.8221994709	-0.2949704937
H	2.6973968145	1.3926056811	0.5826236205
H	1.0598462906	1.2328845858	2.7540399676
H	0.0585115870	-1.2048655219	2.2411511860
H	1.0221168040	1.9443579047	0.3929887345
H	1.2171525053	0.2320899721	-1.3681739495
H	2.0663664349	-0.1816441771	2.3474562795
H	-0.8251033171	-0.1897241564	-0.2174001403
H	-0.7822162450	0.1783552480	1.9617406805
H	-0.2154915465	-3.5053500516	-1.2347510860

zwitterion structure, optimization in the dielectric continuum

C	1.3328765797	0.3717326532	1.9563843671
C	1.6881104223	1.0375065511	0.6333760332
C	1.2428676272	-0.0013581018	-0.4009298516
C	-0.0554478030	-0.5877585804	0.1815114656
N	0.0192769316	-0.2961641313	1.6619125642
C	-0.2047510217	-2.1193530222	0.0036066135
O	-0.2998109611	-2.5198372781	-1.1730755698
O	-0.2009673986	-2.7939052349	1.0682177652
H	2.0002478913	-0.7858047067	-0.5027436325
H	2.7541527288	1.2668483437	0.5734399874

H	1.2132695748	1.0465480536	2.8028566586
H	-0.0692090435	-1.2316313404	2.1040770594
H	1.1273913537	1.9711097598	0.5172530807
H	1.0706632891	0.4286275957	-1.3887729999
H	2.0451593913	-0.4145116833	2.2136533479
H	-0.9365678247	-0.0832323251	-0.2173207632
H	-0.7637427371	0.2792564471	1.9711678741

Exemplary Q-chem 6.0 input for a C 1s spectrum calculation with the MOM method

```
$molecule
0 1
C       1.3328765797     0.3717326532     1.9563843671
C       1.6881104223     1.0375065511     0.6333760332
C       1.2428676272    -0.0013581018    -0.4009298516
C      -0.0554478030    -0.5877585804     0.1815114656
N       0.0192769316    -0.2961641313     1.6619125642
C      -0.2047510217    -2.1193530222     0.0036066135
O      -0.2998109611    -2.5198372781    -1.1730755698
O      -0.2009673986    -2.7939052349     1.0682177652
H       2.0002478913    -0.7858047067    -0.5027436325
H       2.7541527288     1.2668483437     0.5734399874
H       1.2132695748     1.0465480536     2.8028566586
H      -0.0692090435    -1.2316313404     2.1040770594
H       1.1273913537     1.9711097598     0.5172530807
H       1.0706632891     0.4286275957    -1.3887729999
H       2.0451593913    -0.4145116833     2.2136533479
H      -0.9365678247    -0.0832323251    -0.2173207632
H      -0.7637427371     0.2792564471     1.9711678741
$end

$rem
METHOD CAM-B3LYP
BASIS General
THRESH 12
MAX_SCF_CYCLES 129
solvent_method  pcm
PCM_PRINT      1
MEM_TOTAL  192000
$end

$basis
H 0
aug-cc-pVTZ
****
C 0
aug-cc-pCVTZ
****
N 0
aug-cc-pCVTZ
****
O 0
aug-cc-pCVTZ
****
$end
```

```
$pcm
THEORY            IEFPCM
RADII             UFF
vdwScale          1.1
NonEquilibrium
$end

$solvent
Dielectric        78.39
OpticalDielectric 1.78
$end

@@@
$molecule
1 2
READ
$end
$rem
METHOD CAM-B3LYP
BASIS General
THRESH 12
MAX_SCF_CYCLES 129
unrestricted TRUE
mom_start   1
MOM_METHOD IMOM
scf_guess read
solvent_method  pcm
PCM_PRINT      1
MEM_TOTAL  192000
$end

$basis
H 0
aug-cc-pVTZ
****
C 0
aug-cc-pCVTZ
****
N 0
aug-cc-pCVTZ
****
O 0
aug-cc-pCVTZ
****
$end

$pcm
THEORY            IEFPCM
RADII             UFF
vdwScale          1.1
StateSpecific     Marcus
$end

$solvent
Dielectric        78.39
OpticalDielectric 1.78
$end

$occupied
1:31
1:3 5:31
$end
```
